# Development of spirulina for the manufacture and oral delivery of protein therapeutics

**DOI:** 10.1038/s41587-022-01249-7

**Published:** 2022-03-21

**Authors:** Benjamin W. Jester, Hui Zhao, Mesfin Gewe, Thomas Adame, Lisa Perruzza, David T. Bolick, Jan Agosti, Nhi Khuong, Rolf Kuestner, Caitlin Gamble, Kendra Cruickshank, Jeremy Ferrara, Rachelle Lim, Troy Paddock, Colin Brady, Stacey Ertel, Miaohua Zhang, Alex Pollock, Jamie Lee, Jian Xiong, Michael Tasch, Tracy Saveria, David Doughty, Jacob Marshall, Damian Carrieri, Lauren Goetsch, Jason Dang, Nathaniel Sanjaya, David Fletcher, Anissa Martinez, Bryce Kadis, Kristjan Sigmar, Esha Afreen, Tammy Nguyen, Amanda Randolph, Alexandria Taber, Ashley Krzeszowski, Brittney Robinett, David B. Volkin, Fabio Grassi, Richard Guerrant, Ryo Takeuchi, Brian Finrow, Craig Behnke, James Roberts

**Affiliations:** 1 Lumen Bioscience, Seattle, WA USA; 2 Institute for Research in Biomedicine, Faculty of Biomedical Sciences, Università della Svizzera Italiana, Bellinzona, Switzerland; 3 Division of Infectious Diseases & International Health, University of Virginia School of Medicine, Charlottesville, VA USA; 4 Department of Pharmaceutical Chemistry, Vaccine Analytics and Formulation Center, University of Kansas, Lawrence, KS USA; 5 Present Address: Merck & Co., West Point, PA USA; 6 Present Address: Sandia National Laboratories, Livermore, CA USA; 7 Present Address: TScan Therapeutics, Waltham, MA USA

**Keywords:** Genetic engineering, Recombinant protein therapy, Expression systems, Protein delivery

## Abstract

The use of the edible photosynthetic cyanobacterium *Arthrospira platensis* (spirulina) as a biomanufacturing platform has been limited by a lack of genetic tools. Here we report genetic engineering methods for stable, high-level expression of bioactive proteins in spirulina, including large-scale, indoor cultivation and downstream processing methods. Following targeted integration of exogenous genes into the spirulina chromosome (chr), encoded protein biopharmaceuticals can represent as much as 15% of total biomass, require no purification before oral delivery and are stable without refrigeration and protected during gastric transit when encapsulated within dry spirulina. Oral delivery of a spirulina-expressed antibody targeting campylobacter—a major cause of infant mortality in the developing world—prevents disease in mice, and a phase 1 clinical trial demonstrated safety for human administration. Spirulina provides an advantageous system for the manufacture of orally delivered therapeutic proteins by combining the safety of a food-based production host with the accessible genetic manipulation and high productivity of microbial platforms.

## Abstract (web summary)

Spirulina is used to manufacture a therapeutic antibody against campylobacter.

## Main

Modern biotechnology relies on the domestication of cells as biological factories through genetic engineering^1–3^. Expression platforms include *Escherichia coli*, used to manufacture relatively small and simple therapeutic proteins^4^, and yeasts and mammalian cells for more complex molecules^5,6^. Adoption of new expression platforms depends on the availability of methods for genetic manipulation of the organism to achieve stable, high expression of exogenous proteins, and on whether the organism possesses biological traits compatible with large-scale manufacturing and commercialization. Genetically engineered plants have performance characteristics different from cultured cells, such as photosynthetic growth and easy scalability^7–10^. However, their promise has not been realized for reasons including cumbersome genetic methods, slow growth rates, low product yields and regulatory constraints^11–13^. Algae have been considered as alternatives to plants for biotechnology applications^14^, but are difficult to engineer genetically and expression levels of exogenous protein are low and often unstable^15,16^. To date, no biologic therapeutic has been commercialized using an algal platform.

Photosynthetic spirulina is the only microorganism that is commercially farmed worldwide as a food. Its protein content exceeds that of all other food crops^17^, making it a strong candidate for the expression of therapeutic proteins at high levels. Spirulina’s asexual reproduction mitigates the risk of gene escape into the food chain and the associated food security concerns and regulatory burden. Spirulina therefore promises the benefits of plant-based biopharmaceuticals and may overcome the challenges and limitations of other crop- and algal-based platforms.

Here we report our discovery of versatile genetic engineering methods for spirulina that include integration of exogenous genes into the spirulina chromosome by markerless homologous recombination, and stable, high-level expression of therapeutic proteins including bioactive peptides, single-chain antibodies, enzymes, signaling proteins and vaccine antigens. We describe the development of indoor cultivation technology for the large-scale manufacturing of biopharmaceuticals in spirulina under current good manufacturing practices (cGMP). We further report the development of an edible, antibody-based therapeutic targeting gastrointestinal infection by *Campylobacter jejuni* to illustrate application of the spirulina platform to an important unmet medical need, including validation in animal models of campylobacteriosis and demonstration of its safety and pharmacokinetics in a human phase 1 clinical trial.

## Results

### Spirulina is naturally competent for transformation

**Fig. 1:**
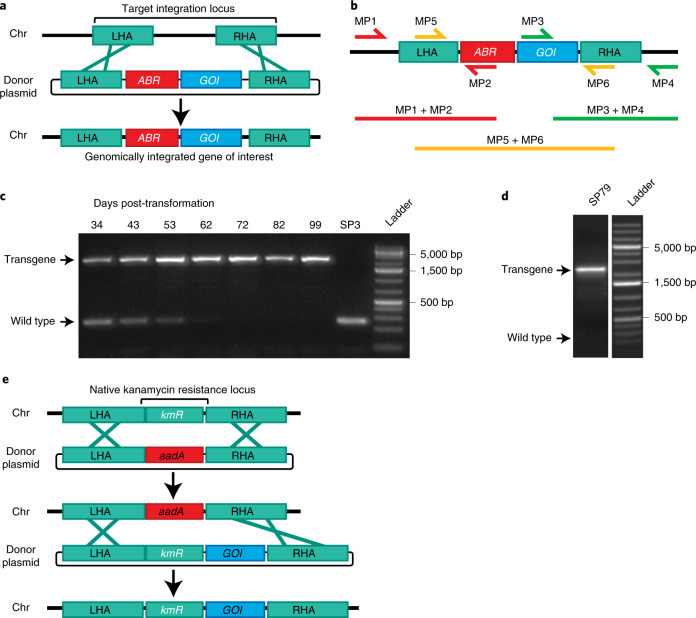
Homologous recombination into the spirulina chromosome. **a**, Plasmid DNA containing an antibiotic-resistance (*ABR*) gene and a gene of interest (*GOI*) flanked by LHA and RHA. A double-crossover inserts *ABR* and *GOI* into the target locus, replacing genomic DNA. **b**, Diagram of primer pairs for PCR genotyping. Amplification of LHA and RHA includes one priming site (MP1 and MP4) present only in the spirulina genome at the target locus. Sanger sequencing of the PCR product of the central primer pair (MP5 + MP6) confirmed faithful integration. **c**, Segregation analysis of strain SP607. Spirulina strain SP3 was transformed on day 0 with donor DNA containing an antibiotic marker and a transgene and cultured under antibiotic selection. Spirulina was collected at the indicated time points, and full transgene products (MP5 + MP6) were amplified from genomic DNA by PCR. Genotyping was performed once. **d**, Long-term transgene stability. Spirulina strain SP79 was genotyped after continuous culture for >3 years. PCR from genomic DNA was performed with primers targeting the full transgene region (MP5 + MP6). Genotyping was performed once. **e**, Strategy of markerless transgene integration (see main text for details).

**Extended Data Fig. 1:**
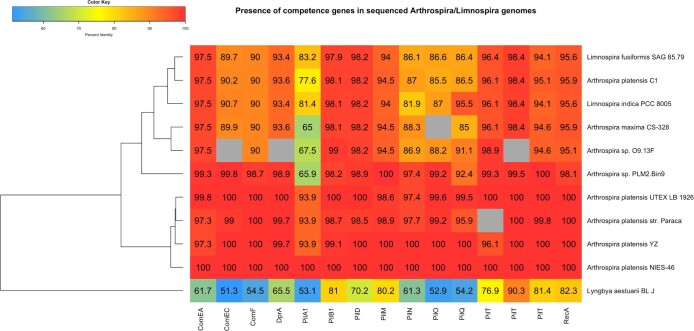
Table of competence genes. The presence of competence genes in sequenced arthrospira/limnospira genomes was determined using reciprocal best hits against the *A. platensis* NIES-39 competence genes previously identified^53^. Genomes were retrieved on GenBank and BLASTp was used to identify reciprocal hits with e-values <1^–5^ and query coverage >50%. Cell labels indicate the percent identity relative to the respective NIES-39 gene; cells in grey indicate that no homolog was identified*. Lyngbya aestuarii* BL J was included as an outgroup.

Our experimental analyses showed that spirulina is naturally competent for transformation, despite being viewed as refractory to genetic manipulation^18,19^. Transformation competence was achieved by cocultivation of spirulina with companion microorganisms (below). Competent spirulina (UTEX LB1926 and NIES-39) were exposed in liquid culture to an integrating DNA vector containing a selectable marker and a gene of interest, flanked on both sides by sequences homologous to the spirulina chromosome. Targeted integration of the transforming DNA by homologous recombination was demonstrated by sequencing of chromosomal DNA using primers flanking the insertion site (Fig. 1a,b). This transformation method yielded a pool of approximately 100 independent transformants, as determined by next-generation sequencing of a culture that had been exposed to a library of barcoded integrating DNA vectors. Consistent with these observations, the 15 genes associated with the pathway for natural competence in cyanobacteria are present as complete open reading frames in most available *Arthrospira* genomes (Extended Data Fig. 1).

**Extended Data Fig. 2:**
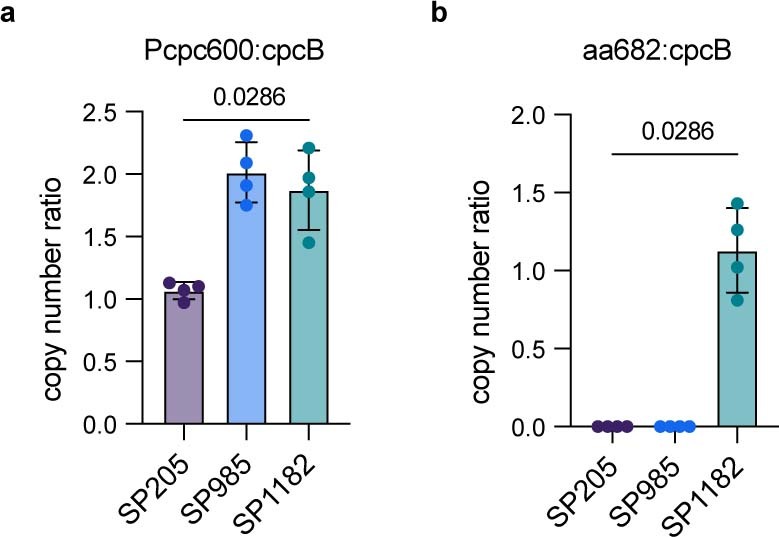
Determination of transgene copy number by qPCR. Specific primer pairs were used in qPCR reactions to detect for the presence of three different sequences. Genomic DNA from spirulina strains SP205 (parental), SP985 (Pcpc600-driven expression of anti-*C. difficile* VHH 5D), and SP1182 (Pcpc600-driven expression of aa682) were used as templates. The gene for cpcB was used as an endogenous reference gene. The cpc600 promoter was expected to be present as a single copy in SP205, while both SP985 and SP1182 contained a second copy for transgene expression. The gene for aa682 should only be present in SP1182. Gene copy number ratio was calculated using the ∆∆Ct method for **a**. cpc600 promoter and *cpcB* and **b**. VHH transgene for aa682 and cpcB. Bars in both figures represents the mean ± SD of four independent replicates. Statistical significance was assessed by a two-tailed Mann-Whitney t-test.

Segregation of the transgene to homozygosity in polyploid spirulina occurred 8–10 weeks after transformation under continuous selection (Fig. 1c). Once homozygous, PCR and DNA sequencing showed that the transgene was genetically stable. No changes in transgene DNA sequence were found in nine of nine strains continuously propagated for at least 1 year, and two of two grown for 3 years (>800 cell generations) (Fig. 1d and Methods). Clonal derivatives were recovered by microisolation of individual spirulina filaments and were verified as containing a single insertion per chromosome by comparison to an endogenous gene using quantitative PCR (qPCR) (Extended Data Fig. 2).

### Induction of spirulina competence

Spirulina UTEX LB1926 (UTEX culture collection) was not axenic, but microisolation of single filaments was used to prepare axenic strains. Eight of 11 single filaments produced axenic cultures, as verified by microscopic examination and confirmed by cultivation on lysogeny broth (LB) + Spirulina-Ogawa-Terui (SOT) media plates. When exposed to two different integrating DNA vectors, the three xenic filament-derived strains—but none of the eight axenic strains—were transformable, suggesting that competence for transformation is associated with the presence of other microorganisms.

Two microorganisms in the original xenic UTEX LB1926 culture were clonally isolated and identified as belonging to the genera *Sphingomonas* and *Microcella* (Supplementary Table 1). Twelve individual colonies were cocultured in liquid medium with axenic UTEX LB1926. The axenic spirulina strain remained nontransformable and all 12 cocultures became competent for spirulina transformation. Similar results were obtained for NIES-39. Therefore, we used xenic strains for genetic transformation and derived axenic strains for subsequent protein production.

### A markerless method for engineering of spirulina

The site of integration was determined by 1.0–1.5-kb homology arms flanking the transforming genes. A single homology arm was ineffective, indicating that integration occurs by double crossover, as is typical for natural transformation^20^. Six of the 11 tested sites of integration accommodated an insertion with no associated growth deficit.

One insertion site corresponded to NCBI reference sequence *NIES39_RS07765*, which codes for a protein homologous to kanamycin aminoglycoside acetyltransferases (hereafter referred to as KmR). It conferred kanamycin resistance in *E. coli*, and its deletion rendered spirulina kanamycin sensitive. This allowed development of a markerless engineering strategy by replacement of the *KmR* gene with an exogenous streptomycin resistance gene (*aadA*), followed by replacement of the exogenous *aadA* gene with a tandem gene cassette comprising the *KmR* gene and the gene of interest. This strain contained the gene of interest integrated adjacent to the *KmR* gene and no other exogenous DNA (Fig. 1e).

**Extended Data Fig. 3:**
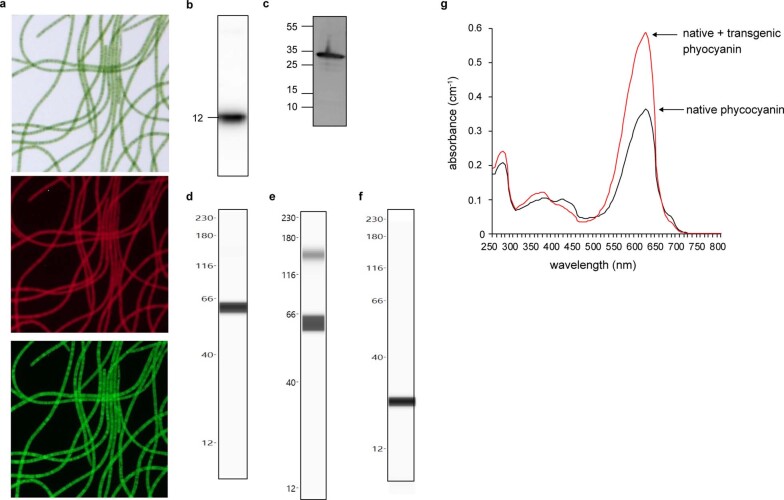
Expression of representative transgenic proteins in spirulina. Uniform expression of GFP in a population of spirulina transformed with a transgene (SP699). Shown are a bright field image (top), and fluorescence images of chlorophyll (middle) and GFP (bottom). **b**. Western blot of spirulina expressing a 12 kDa orally active and antibacterial peptide, MAA (mammary associated amyloid) (SP13); peptide represents 4-5% of cell protein. **c**. Western blot of spirulina expressing a vaccine antigen, a repeat domain of malaria *P. yoelii* circumsporozoite (CSP) protein fused to with core protein of the woodchuck hepadnavirus (WHcAg) (SP82), which self-assembles into a multivalent virus-like particle; antigen represents 4% of cell protein. **d**. CEIA of spirulina expressing a single-domain antibody, an anti-SARS-CoV-2 VHH fused to a self-dimerizing scaffold (SP1741); VHH represents 9.3% of cell protein. **e**. CEIA of spirulina expressing a single-domain antibody, an anti-SARS-CoV-2 VHH fused to a self-dimerizing scaffold (SP1825); VHH represents 29.8% of cell protein. **f**. CEIA of spirulina expressing an enzyme, the catalytic domain from a *C. difficile*-specific, bacteriophage-derived endolysin (SP1287); enzyme represents 0.54% of cell protein. **g**. Spectroscopic analysis of spirulina lysates containing a transgenic pigment protein (SP84); pigment protein represents 10% of cell protein. Phycocyanin, a natural blue pigment protein present in wild type spirulina, was overexpressed with a transgenic copy of the native gene. Absorbance spectra for wild-type (black) and transgenic (red) spirulina are shown. Each experiment was performed once.

We introduced a variety of exogenous DNA vectors into the spirulina genome, including single genes, tandem genes, operons and sequential engineering of different insertion sites (Supplementary Table 2). The largest transforming DNA cassette was 6.0 kb and contained a seven-gene C-phycocyanin operon. Exogenous proteins were expressed uniformly in spirulina filaments (Extended Data Fig. 3). We demonstrated stable intracellular expression of diverse exogenous proteins, including bioactive peptides, antigen-binding domains (VHHs), protein pigments and enzymes (Extended Data Fig. 3). Proteins expressed using a strong, constitutive promoter from the C-phycocyanin locus (Pcpc600) accumulated to represent up to 29% of total soluble protein (Extended Data Fig. 3e).

### Expression of single-chain antibody fragments in spirulina

**Fig. 2:**
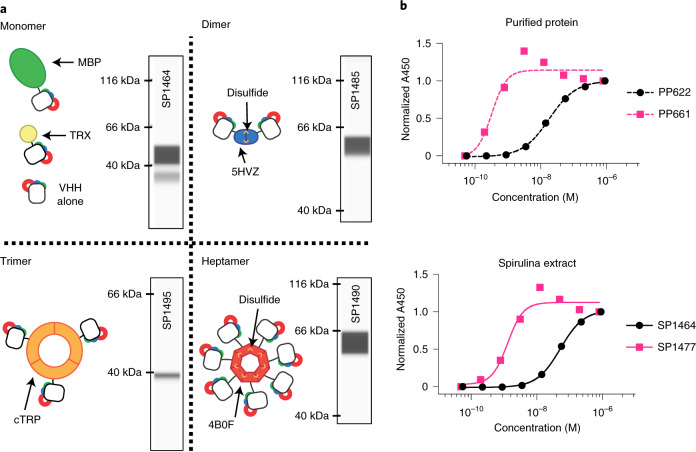
VHH scaffolding strategies. **a**, Cartoons of multimeric scaffolds with sample expression data for VHHs in spirulina. Monomeric (MBP and thioredoxin (TRX)), dimeric (5HVZ), trimeric (cTRP) and heptameric (4B0F) scaffolding proteins were used to multimerize VHHs expressed in spirulina. Intersubunit disulfides confer additional stability to the dimeric and heptameric scaffolds, and these forms were commonly expressed with an MBP tag to improve solubilization. Inset CEIA blots for each scaffold demonstrate spirulina expression of a SARS-CoV-2 RBD-binding VHH^49^ fused to the indicated scaffolding protein. CEIA analysis was performed performed once for each strain. All proteins were observed at the appropriate size. **b**, Increase in apparent binding activity by dimerization of VHHs, as measured by ELISA with purified VHH (top) and spirulina extract (bottom). *E. coli*-expressed and purified monomeric (PP622) and dimeric (PP661) forms of RBD-binding VHH were assayed with RBD and compared with the binding activity of identical proteins present in spirulina extracts (SP1464 and SP1477, respectively). The concentration of VHH in the spirulina extracts was determined by CEIA. ELISA was performed once with duplicate samples. Absorbance was normalized to the highest absorbance within each sample. EC_50_ values of 18.3 and 52.3 nM were recorded for monomeric VHH in purified (PP622) and extract (SP1464) samples, respectively, while those for dimeric VHH were 0.32 and 1.29 nM for the purified (PP661) and extracted forms (SP1477), respectively.

**Extended Data Fig. 4:**
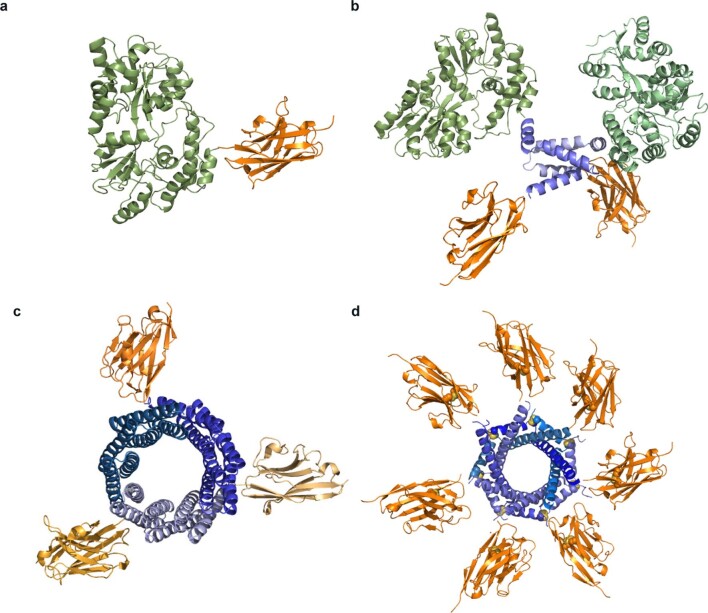
Model representations of heterologous proteins designed for expression in spirulina. a. Ribbon representation of a monomeric VHH (orange; PDB ID:6WAQ)^54^ with the solubility enhancer, MBP (green; PDB ID: 5M13)^55^. The mature, folded protein results in a monomeric VHH as a fusion to MBP and a C-termini 6X-his affinity tag. b. Ribbon representation of a VHH (orange) with a dimerization motif (blue; PDB ID: 5HVZ)^56^ and the solubility enhancer, MBP (green). The mature, folded protein results in a dimeric VHH where dimerization is facilitated by the disulfide-linked dimerization motif. The single polypeptide also contains the solubility enhancer MBP and C-terminal 6X-his affinity tag. c. Ribbon representation of a trimeric VHH (orange). The mature, folded protein results in trimeric VHH (orange) where trimerization is facilitated by the self-assembling homotrimer t-cTRP9X_3_ (blue)^57^. The single polypeptide also contains a C-terminal 6X-his affinity tag. d. Ribbon representation of heptameric VHH (orange) with the heptamerization motif (blue; PDB ID: 4B0F)^58^. The mature, folded protein results in a heptameric VHH where heptamerization is due to intrachain disulfide bond between individual protomers. The polypeptide also contains an N-terminal solubility enhancer MBP fusion and C-terminal 6X-his affinity tag. All structures generated using Pymol (Schrodinger).

**Extended Data Fig. 5:**
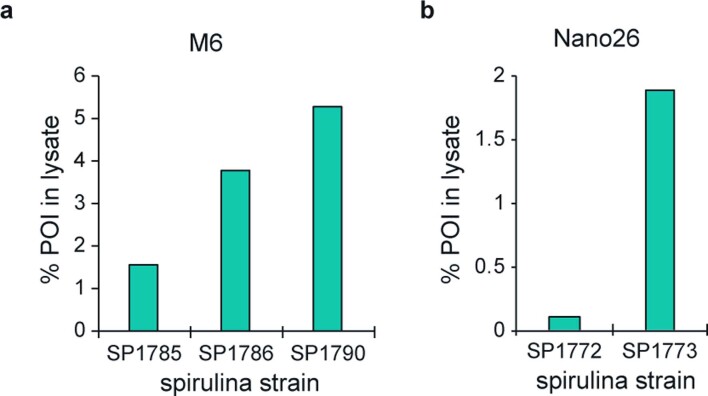
Effect of MBP fusion on VHH expression in spirulina. CEIA was used to evaluate the relative expression level of fusions of two norovirus-binding VHHs in spirulina: a) M6 (ref. ^59^) and b) Nano26 (ref. ^60^) The VHH M6 was expressed with no fusion (SP1785), MBP fused to the C-terminus (SP1786), or MBP fused to the N-terminus (SP1790). The VHH Nano26 was expressed with no fusion (SP1772) or MBP fused to the C-terminus (SP1773). All proteins contained a C-terminal His-tag. Clarified lysates from the indicated spirulina strains were normalized by total soluble protein, run on a Jess system, and the protein of interest (POI) detected with an anti-His-tag antibody. The POI was quantified with a purified His-tagged reference standard and the percentage in lysate calculated relative to total soluble protein. Results represents a single measurement for each strain.

**Extended Data Fig. 6:**
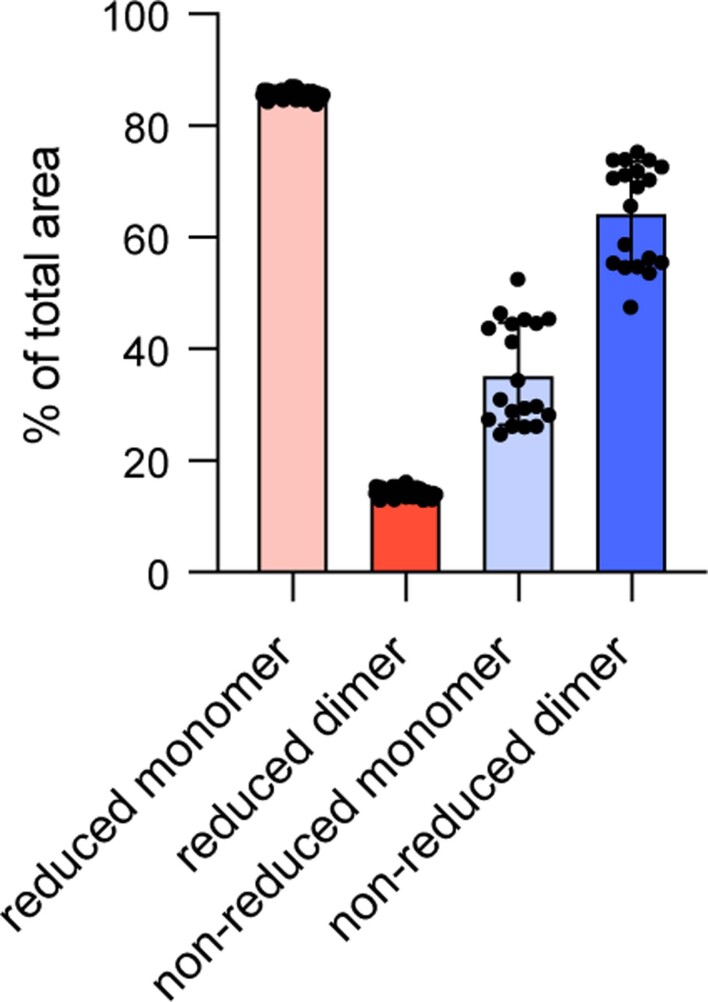
CEIA analysis of the oxidation state of disulfides in dimeric proteins expressed in spirulina strain SP1313. Strain SP1313 expresses the anti-tcdB VHH 7F^61^ fused to MBP through the dimerization domain 5HVZ. The dimeric form of the protein should have one disulfide within each VHH and two disulfides to maintain association between 5HVZ subunits. Relative percentage of monomer and dimer peak areas observed under reducing and non-reducing conditions is shown. Protein was detected with an anti-His primary antibody followed by an anti-mouse secondary NIR antibody. SP1313 lysate was run in reducing conditions with DTT for 6 independent experiments with 3 lysates assessed in each run, and in non-reducing conditions for 5 independent experiments with 2 lysates assessed in each run. Each data point represents a peak area as quantified by Protein Simple’s SW Compass Software. Bars indicate mean ± SD. Four representative spirulina strains expressing different VHHs on the 5HVZ scaffold have been analyzed in this manner, and the portion of scaffolded VHH in the dimeric state in non-reduced samples ranged from 50 to 100%.

Antigen-binding domains (VHHs) from camelid single-chain antibodies are expressible in prokaryotes like spirulina^21^. Intracellular VHHs were constitutively expressed in various formats, including monomers, dimers, trimers and heptamers (Fig. 2a and Extended Data Fig. 4). Multimers routinely demonstrated subnanomolar apparent *K*_D_ levels (Fig. 2b). Expression of VHHs as high-avidity multimers could bypass a need for affinity maturation and may accelerate product development. Monomeric VHHs were typically expressed as a fusion protein with a chaperone, such as the *E. coli* maltose-binding protein (MBP), to increase expression levels (Extended Data Fig. 5). Disulfide bonds involved in the assembly of the dimeric VHH scaffold formed at 50–100% efficiency (Extended Data Fig. 6). Described here are spirulina strains with constitutive VHH expression levels of 0.7, 3.4, 4.0, 4.3, 9.3 and 29% of soluble protein (Fig. 2 and Extended Data Fig. 3).

### Prevention of campylobacter disease

Enteric infectious diseases are designated as high-priority antimicrobial resistance threats by the Centers for Disease Control and Prevention, adding urgency to the search for new therapeutic tools. Diarrhea accounts for 10% of the 7.6 million annual deaths in children under the age of 5 years^22^. *Campylobacter jejuni* is among the most common causes of bacterial gastroenteritis and is a leading cause of infant mortality in the developing world^23–25^.

**Fig. 3:**
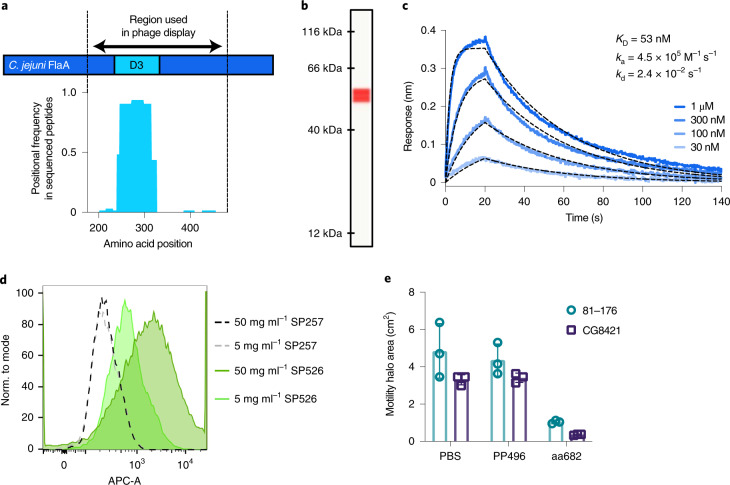
Characterization of spirulina-expressed, anti-campylobacter VHH. **a**, Epitope mapping of VHH interaction with FlaA. Peptides derived from the D2/D3/D4 region of FlaA (amino acids 177–482) were panned by phage display. Enriched clones were sequenced after two or three rounds of panning. Results represent average positional frequency observed in two independent panning experiments. **b**, CEIA quantification of aa682 expressed in SP1182. Clarified lysate from SP1182 was displayed on a Jess system, with an anti-His-tag antibody used for detection. A single peak was observed at the predicted MW of 54.8 kDa. Using a standard curve of purified protein (Extended Data Fig. 7), the amount of soluble aa682 was ~3% of total biomass. Result is representative of dozens of independent experiments. **c**, Binding kinetics of spirulina-expressed aa682 with recombinant FlaA measured by BLI. Streptavidin-coated biosensors were loaded with biotinylated FlaA, and association and dissociation were measured. Curve fitting was performed using a 1:1 binding model. **d**, Binding of VHH to intact *C. jejuni*. Soluble extracts from spray-dried spirulina biomass containing an irrelevant VHH (SP257) or FlagV6-MBP (SP526) were incubated with *C. jejuni* 81–176 and stained with a fluorescently labelled anti-His-tag antibody. Fluorescence was measured in the allophycocyanin channel (APC-A) by flow cytometry. **e**, Inhibition of *C. jejuni* motility by aa682. Two strains of *C. jejuni* (81–176 and CG8421) were grown on soft agar plates in the presence of aa682 or an irrelevant VHH control (PP496). Halo areas (mean ± s.d.) were measured for triplicate samples at either 40 h (81–176) or 66 h (CG8421) after plating.

The VHH FlagV6 binds to flagellin (FlaA), a subunit of *C. jejuni* flagella^26^. Its binding site was mapped to the D3 domain of FlaA by phage display of peptides tiled across the FlaA protein (Fig. 3a). Spirulina strain SP526 expressed a monovalent fusion of FlagV6 VHH and MBP, which bound to recombinant FlaA with a *K*_D_ of 53 nM and was constitutively expressed in the cell cytoplasm at 3% of dry biomass (equivalent to 8% of soluble protein) (Fig. 3b,c).

The D3 domain of FlaA is surface accessible for VHH binding^27^. Specific binding of FlagV6-MBP to intact *C. jejuni* flagella was demonstrated by flow cytometry. Aqueous extracts of SP526 were incubated with live *C. jejuni* 81–176 and then stained with anti-His-tag antibody. Binding to *C. jejuni* was compared with an extract from SP257, which expressed an irrelevant VHH (Fig. 3d). The major flagellin protein (FlaA) is required for motility, and motility is required for virulence^28^. Binding of VHHs to FlaA has been shown to prevent campylobacter motility in vitro^26^. FlagV6-MBP blocked the motility of *C. jejuni* (Fig. 3e) and was therefore predicted to inhibit pathogenesis in vivo.

**Fig. 4:**
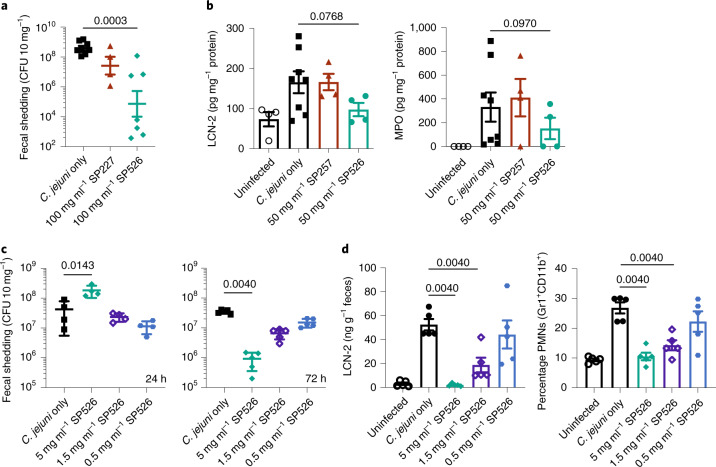
Prevention of *C. jejuni* infection in mice. **a**, Fecal shedding of *C. jejuni* in a mouse model of infection. Mice received a single 200-μl dose of spirulina strain SP227 (no VHH, *n* = 4), strain SP526 (analog of aa682, *n* = 7) or vehicle (*n* = 8) on days –1, 0, 1, 2 and 3 relative to challenge. Bacterial shedding in stool (CFU 10 mg^–1^ feces) was measured 7 days after challenge. **b**, Fecal biomarkers of inflammation (LCN-2 and MPO) measured 11 days after infection. Mice received a single 200-μl dose of spirulina strain SP257 (irrelevant VHH, *n* = 4), strain SP526 (*n* = 4) or vehicle (*n* = 8) on days –1, 0 or 1 relative to challenge. Uninfected mice were treated with vehicle (*n* = 4). **c**, Fecal shedding after treatment with a single dose of SP526. Mice received a single 400-μl dose of spirulina resuspension or vehicle 1.5 h before challenge with *C. jejuni*. Bacterial shedding in stool was measured 24 h (*n* = 4 per group) and 72 h (*n* = 5 per group) after challenge. **d**, Fecal biomarkers of inflammation (LCN-2 and PMNs) measured 72 h after challenge and treatment with a single dose of SP526 (*n* = 5 per group). All data represented as mean ± s.e.m.. A one-tailed Mann–Whitney test was applied to assess statistical significance between cohorts, with *P* values indicated. Each animal experiment was performed once.

Mouse models were used to test whether orally delivered spirulina containing an anti-campylobacter VHH could prevent enteric campylobacter infection^29^. Mice were challenged on day 0 with 10^6^ colony-forming units (CFU) of *C. jejuni* 81–176 by oral gavage. The experimental group received 10 mg of SP526 (containing 300 µg of FlagV6-MBP) by oral gavage 1 h before campylobacter challenge, and on days 1 and 2. Control groups were either untreated or treated with 10 mg of spirulina containing either an irrelevant recombinant protein or an irrelevant VHH. Treatment with SP526 reduced campylobacter fecal shedding by three to four orders of magnitude and significantly reduced two biomarkers of intestinal inflammation, lipocalin-2 (LCN-2) and myeloperoxidase (MPO) (Fig. 4a,b). Diarrhea was observed in all mice in the control groups, but in none of the mice receiving SP526.

A second experiment used a challenge dose of 10^8^ CFU of *C. jejuni* 81–176. Dose-ranging experiments showed that a single prophylactic oral dose of 2 mg of dry SP526 biomass (60 µg of FlagV6-MBP; final concentration of approximately 10^–6^ mol l^–1^ mouse small intestine^30^) prevented campylobacter disease, as measured by stool LCN-2 and myeloid cell infiltration of the cecum. Furthermore, this accelerated campylobacter expulsion from the gut 24 h after challenge and reduced campylobacter shedding at 72 h (Fig. 4c,d).

### Large-scale continuous growth

Spirulina is cultivated in open ponds at commercial scale, but uncontrolled exposure to environmental contaminants makes this unsuitable for the manufacture of biopharmaceuticals under FDA cGMP. Therefore, we developed modular, indoor, 160–2,000-l, vertical, flat-panel photobioreactors that were pH controlled and air-mixed. Commercial-scale manufacturing is accomplished by constructing arrays of these reactors rather than by constructing larger reactors.

An advantage of this platform is the simplicity of large-scale growth compared with traditional fermentation platforms. Spirulina thrives at pH> 10 and high total salinity, and grows without a carbon-based source of energy. These allowed the use of unsealed reactors under sanitary, but not aseptic, conditions. Single-use polyethylene (mylar) bags contained the spirulina culture, eliminating sterilization downtime, which is one of the bigger bottlenecks in biopharmaceutical processes^31^.

**Extended Data Fig. 8:**
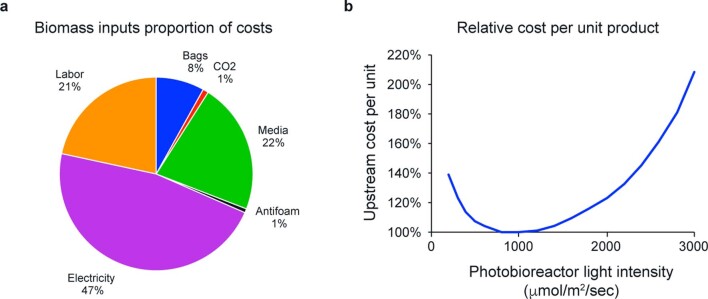
Cost optimization. **a**. Cost components of cGMP biomass production. **b**. Spirulina productivity is a function of light intensity and is empirically determined in the described system with SP1182 as the production organism and with current operating parameters. Cost per unit biomass includes labor, capitalized cost of operating lighting system (varies by light intensity), and capitalized costs of other upstream components (independent of light intensity). Minimal cost per unit biomass was achieved at a light intensity of approximately 100 μmol/m^2^/sec.

Energy for illumination with full-spectrum, adjustable-intensity light-emitting diodes (LEDs) was the major component of production cost (Extended Data Fig. 8a). The complex relationship between the capital and operational costs of biomass growth was evaluated as a function of light intensity, and an optimum that achieved the greatest productivity per unit energy cost was identified (Extended Data Fig. 8b).

Cultures were maintained without antibiotic selection for sequential 1-week growth cycles. Growth rates of photosynthetic microbes differ from those of heterotrophic microbes, because growth is light limited. At low densities spirulina can undergo exponential growth with a doubling time of 2–3 h. At densities greater than ~0.5 g l^–1^, growth becomes light limited and growth rates are linear (that is, a constant amount of biomass l^–1^ d^–1^). On a weekly basis, cell densities reached ~4 g l^–1^ and the biomass was harvested by passage over a series of stainless steel screens. A portion of the resulting slurry was used to reinoculate the reactors, the remainder being processed into drug product.

### Simple downstream processing

**Extended Data Fig. 9:**
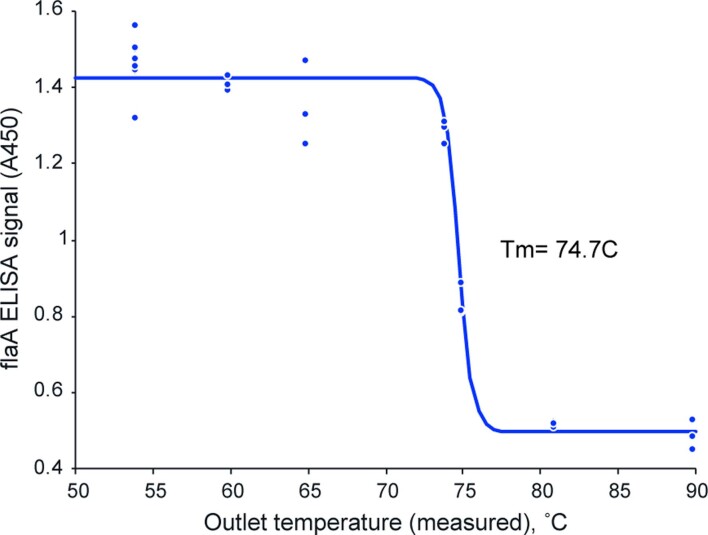
VHH stability during spray drying. FlaA binding activity of aa682 in biomass versus drying temperature. Biomass of strain SP1182 was dried across a range of temperatures, extracted at 10 mg/ml biomass, and the extracts were diluted to a constant 0.039 mg/ml assay concentration. Binding activity of the extracts to FlaA was measured by ELISA. Binding activity was unaffected by drying temperatures <73 °C.

The spirulina slurry was rinsed with a trehalose solution and then spray-dried. A large parameter space was evaluated for efficiency of drying, moisture content and retention of antibody activity. Conditions were identified (Extended Data Fig. 9) in which suitable system efficiency was achieved while maintaining >90% of antibody activity. The process was translated to a larger-scale (5 kg h^–1^) spray-dryer equipped with a centrifugal atomization system. With minor optimization of throughput, inlet and outlet temperatures and air flow rates, equivalent dryer performance at a ~20×, single-step scale-up was achieved.

**Fig. 5:**
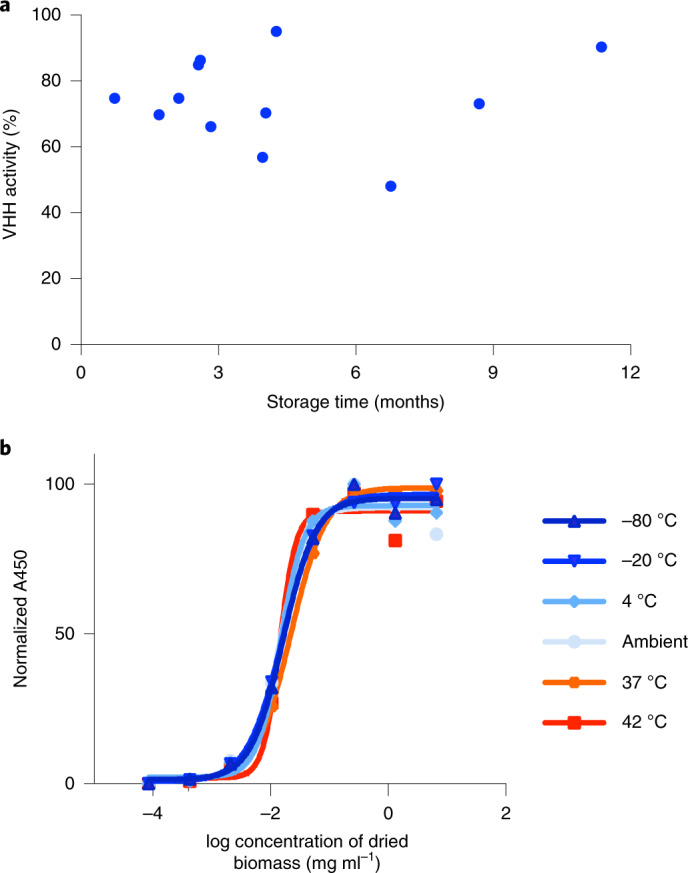
Stability testing of SP1182 binding activity. **a**, Batches of spray-dried SP1182 were stored at room temperature for the indicated lengths of time, and binding activity was assessed by ELISA. Purified aa682 binding to recombinant FlaA was used to generate a standard curve by linear regression. VHH activity calculated for spirulina samples was normalized to 100% assuming an expression level of 3% aa682 per unit of biomass. Each point represents a different batch of biomass, presented as the mean of two technical replicates. **b**, VHH binding activity of SP526 stored at a range of temperatures over 6 months. Aliquots of biomass were prepared in duplicate and stored at the indicated temperature with desiccant. Extracts prepared from spray-dried biomass were serially diluted and assessed for binding activity to recombinant FlaA by ELISA. Each experiment was performed once.

The dry powder was collected and sealed in light- and moisture-proof packaging; antibody activity was retained at up to 42 °C for at least 6 months (Fig. 5). Packaging the powder into vegetarian capsules was the final downstream process. The manufacturing system used was cGMP, as regulated under 21 CFR Parts 210/211. Product quality specifications, including bioburden and elemental impurities, conformed to industry standards for oral solid-dose therapeutics including United States Pharmacopeia (USP) <1111> and USP <232>.

### Gastrointestinal delivery

**Fig. 6:**
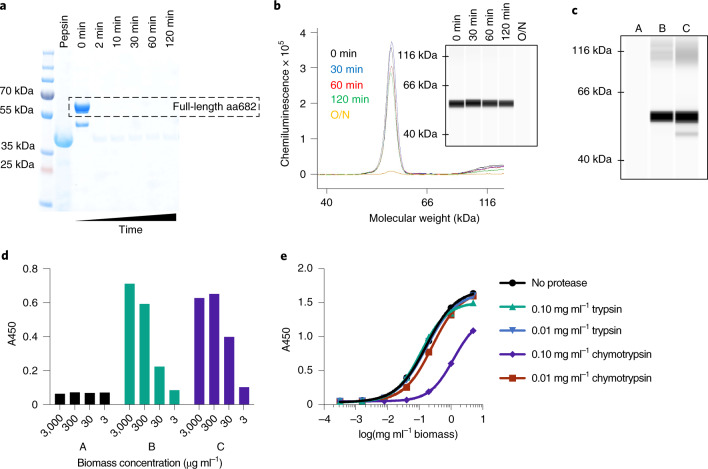
Protease sensitivity of aa682. **a**, SDS–PAGE analysis of purified aa682 incubated with simulated gastric fluid supplemented with 2,000 U ml^–1^ pepsin. Results are representative of two independent experiments. **b**, CEIA of pepsin-digested spirulina biomass resuspension. Dried spirulina biomass of SP1182 was resuspended in simulated gastric buffer and incubated with pepsin for 0–120 min or overnight (O/N). Whole biomass samples were denatured and analyzed on a Jess system. Recombinant aa682 was detected with an anti-His-tag antibody, and data are representative of four independent experiments. **c**, CEIA of spirulina biomass extracted under different conditions. Dried spirulina biomass of SP1182 was resuspended in simulated gastric buffer (pH 3.0, no pepsin) and the presence of aa682 in buffer was analyzed (A). The biomass was first incubated (A), brought to intestinal pH (pH >5.0) (B) then compared with aa682 extraction by direct biomass resuspension in bicarbonate buffer (pH >7.0) (C). Data are representative of two independent experiments. **d**, ELISA-based, antigen-binding analysis of spirulina lysates prepared as in **c** assayed for binding to recombinant FlaA. Samples B and C yielded approximate EC_50_ binding values of 85.8 and 29.2 μg ml^–1^ biomass, respectively. Data are average of two replicates. **e**, ELISA binding activity of aa682 after in vitro exposure to intestinal proteases. Lysates from SP1182 were incubated with trypsin or chymotrypsin for 1 h. After protease neutralization, aa682 binding activity to recombinant FlaA was measured by ELISA. Data are average of two replicates. Unless noted otherwise, experiments were performed once.

Following ingestion, biologics are initially subjected to the low-pH, high-pepsin gastric environment. aa682 is a version of FlagV6-MBP containing two alterations in the N-terminal residues of the framework region that increase resistance to chymotrypsin^32^. Purified aa682 was fully degraded within 2 min of incubation under simulated gastric conditions (Fig. 6a). However, when delivered within dry spirulina biomass, >70% of aa682 remained intact after 2 h of incubation under gastric conditions (Fig. 6b). Therefore, bioencapsulation of therapeutic proteins within dry spirulina biomass provides protection during gastric transit. Transition of biomass to the higher pH of a simulated duodenal environment was sufficient to extract >90% of encapsulated aa682 from spirulina within 60 min (Fig. 6c). The binding activity of aa682 extracted under duodenal conditions was minimally affected by previous incubation of the biomass under the gastric environment (Fig. 6d).

In vivo efficacy is also dependent upon the sensitivities of therapeutic proteins to intestinal proteases, especially trypsin and chymotrypsin. Protein aa682 was resistant to constitutive intestinal levels of both trypsin and chymotrypsin. Some sensitivity of aa682 to chymotrypsin, but not trypsin, was evident when it was exposed to the higher protease levels that are present immediately after a meal^33^ (Fig. 6e).

### First-in-human clinical safety trial

**Extended Data Fig. 10:**
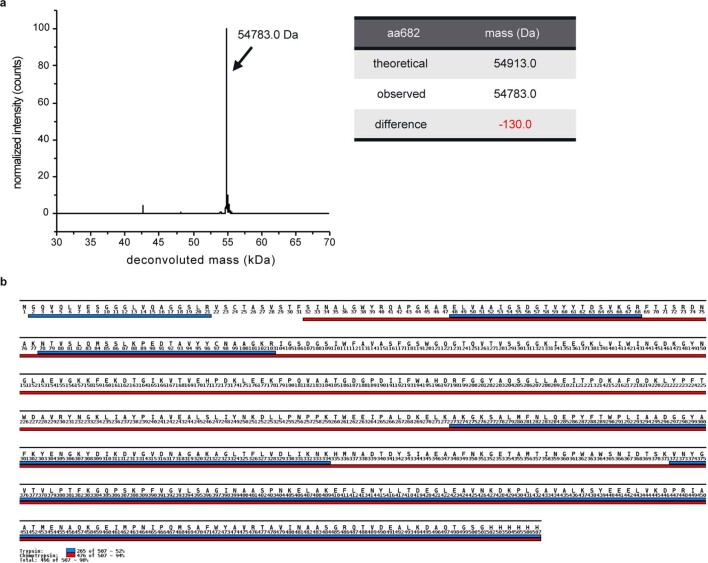
Mass spectrometry analysis of purified aa682. **a**. Intact mass spectrum of aa682 measured by LC-MS. The theoretical mass was 54913 Da, with a measured mass of 54783 Da. The difference in mass would be consistent with loss of the N-terminal methionine. **b**. LC-MS peptide mapping of aa682. Denatured aa682 was digested with trypsin and chymotrypsin, with the resulting peptide fragments characterized by LC-MS. Trypsin and chymotrypsin peptide fragments mapping to aa682 are indicated below the sequence of aa682 in blue and red respectively. Tryptic peptides could be mapped to 52% of the full-length, while chymotryptic peptides mapping to 94% of aa682. Together, the peptide mapping covered 98% of the expected sequence for aa682.

SP1182, a markerless strain expressing aa682, was constructed for human clinical testing. The molecular weight and amino acid sequence of purified aa682 were confirmed by liquid chromatography–mass spectrometry (LC–MS). The measured intact mass matched the theoretical mass of full-length protein, minus post-translational removal of the N-terminal methionine. The amino sequence of aa682 was confirmed by proteolyzed peptide fragment analysis, with 98% coverage of the expected protein sequence (Extended Data Fig. 10). Strains in human clinical testing, including SP1182, were banked as frozen vials, and the DNA sequence of the transgene was confirmed each time a vial was taken from the bank and used to manufacture drug product.

SP1182 and wild-type strain SP3 were cultured in large-scale bioreactors under cGMP conditions and used to formulate the drug product LMN-101 and placebo, respectively. A phase 1 clinical trial in healthy volunteers aged 18–50 years assessed the safety and tolerability of LMN-101. Twenty subjects were randomized to active or placebo treatment with doses up to 3,000 mg of biomass three times daily. LMN-101 was safe and well tolerated. There were no major adverse events or laboratory abnormalities reported during or following the trial. Adverse events were grade 1, were deemed unlikely to be related to the therapeutic and were similar in frequency between experimental and placebo groups (Supplementary Table 3). Measurements of serum VHH levels showed that no absorption had occurred, which was expected for this protein when delivered orally.

## Discussion

The efficacy of oral biologics in the treatment of human disease was first demonstrated for antibody therapy of *E. coli* infection in human infants^34^. Additional successful human clinical trials against rotavirus and *Clostridium difficile* have been reported^35–38^. While these studies established that orally delivered antibodies can be effective, commercial development has suffered from reliance on production systems—such as the milk of hyperimmunized cows—that have proven difficult to scale cost effectively while maintaining FDA-grade lot-to-lot consistency.

Food-based systems for the manufacture of biologics would be ideally suited for this purpose, and some progress has been made with rice-based expression of antimicrobial therapeutics^39^. Here we report our discovery of methods that allow constitutive and stable expression of protein therapeutics in spirulina, with productivities (g therapeutic g^–1^ biomass d^–1^) and potencies (g therapeutic g^–1^ biomass) that surpass, by tens to hundreds of fold, what can be achieved in other food-based platforms^40,41^. Isolation of seed, where plant recombinant proteins are usually expressed, increases apparent potencies but these still fall short of potencies achievable in whole, unfractionated spirulina biomass. Low productivity and potency in plants not only amplify production costs but may also necessitate expensive downstream processing, which is reported to be a weakness of plant-based production systems^11^.

Based on the observations reported here, the repertoire of therapeutics expressible in the spirulina platform would include bioactive peptides, antibody fragments (for example, VHHs), enzymes, antioxidants, signaling proteins (for example, hormones, cytokines) and vaccine antigens, as well as metabolic products of biosynthetic pathways. Like other prokaryotes, however, spirulina can neither recapitulate human protein glycosylation patterns nor efficiently express proteins containing multiple disulfide bonds. Therefore, the subset of therapeutic proteins requiring these for activity would be excluded. It is therefore useful to compare the productivity of the spirulina platform for expression of antibody fragments (VHHs) with that of Chinese hamster ovary (CHO) cells for full-length antibodies. VHH volumetric productivity in spirulina is approximately 25–50 versus 500 mg antibody l^–1^ d^–1^ in CHO cells^42^. Process development of spirulina VHH production is in its infancy, and current volumetric productivity is similar to antibody production in CHO when that was introduced as a production platform^43^. Nevertheless, the materials required for spirulina cultivation—open-topped, LED-illuminated, steel-framed reactors containing single-use disposable bags with a minimal salt growth medium—are considerably simpler than the aseptic fermentation reactors and complex growth media required for production of biologics in CHO. Moreover, the safety of spirulina as an expression host further simplifies the drug manufacturing process. Spirulina hold FDA Generally Recognized as Safe status^44^. The toxicology profile has been positively reviewed by the FDA, and clinical trials have established its safety in both adult^45,46^ and pediatric populations^47^. Consequently, therapeutics produced in spirulina do not need to be purified before oral delivery. Because the manufacturing pipeline is comprised solely of growing and drying spirulina biomass, the technical challenges, operational expenses, in-process controls and safety tests associated with downstream drug processing are either reduced or eliminated. Especially for oral delivery, the simplification of both equipment and upstream and downstream processes can more than offset the benefit apparent from a comparison of volumetric productivities.

These considerations suggest that the spirulina platform may present an opportunity to use biologics not just for treatment but also for prevention of prevalent diseases. Biologics are traditionally produced in small quantities and priced at amounts per dose that make their widespread prophylactic administration almost inconceivable. In contrast, prophylaxis using the spirulina platform could be affordable, even in the developing world. Moreover, refrigerated distribution and intravenous infusion requirements impose further constraints on the deployment of traditional biologics. VHHs and other biologics in dry spirulina powder are shelf stable without refrigeration, facilitating distribution, especially into regions lacking high-quality infrastructure.

Targeted delivery of a therapeutic to its site of action is a key consideration for efficacy. Systemic therapeutics have off-target toxicities, especially at higher doses, and have low partitioning coefficients into mucosal tissues that reduce the amount of active product at the site of action. These are often reasons for clinical failure and a major driver of the high cost of new drug development^48^. Direct delivery of a nonabsorbed therapeutic to the intestine reduces these concerns.

Beyond the example of infectious diseases, current programs in inflammatory bowel diseases, metabolic diseases and oral vaccines illustrate the broad applicability of the spirulina platform; its advantages may also apply to other topical and mucosal surfaces, including upper airway delivery. Perhaps the greatest impact may be with multicomponent biologic cocktails. Cocktails comprised of ten or more therapeutic proteins are in development, whereas therapeutics comprising more than two biologics are generally impractical using conventional platforms due to the complexity of cell line development, manufacturing costs and compounding toxicities of systemic therapeutics. Therapeutic cocktails may be transformative for treatment of complex diseases with multiple underlying pathological processes, and for treatment of pathogens commonly exhibiting therapy-evading genetic variation.

## Methods

### Small-scale spirulina culture

Spirulina strains were grown in liquid culture using SOT medium. For antibiotic selection, medium was supplemented with 70–100 μg ml^–1^ kanamycin or 2.5–5.0 μg ml^–1^ streptomycin. Culture volumes ranged from 3 to 100 ml. In preparation of strains for transformation or downstream processing, cultures were grown in Multitron incubators at 35 °C, 0.5% CO_2_, 110–150 μEi of light and shaking at 120–270 r.p.m., depending on culture volume. Long-term cultures were maintained by incubation in Innova incubators at 30 °C, atmospheric CO_2_, 50–110 μEi of light and shaking at 120 r.p.m.

### Design of integrating vectors for spirulina transformation

For each genomic locus targeted for integration, PCR primers with a 18–20-base-pair (bp) overlapping sequence and a vector backbone were designed to amplify 1.0–1.5-kb DNA fragments from the 5'- and 3'-regions flanking the locus. These regions represented the left homology arm (LHA) and right homology arm (RHA), respectively. Gel-purified fragments were assembled with the linearized backbone vector, which contained a p15 origin and an *E. coli* ampicillin resistance marker, by Gibson assembly. Markers for antibiotic resistance in spirulina were cloned between the two homology arms of the plasmid.

### Transformation of spirulina

Spirulina cultures were grown for 3 days in Innova to optical density (OD_700_ ml^–1^) 0.5–1.0. A cell volume of 50 ml was harvested by centrifugation for 10 min at 1,600× relative centrifugal force (RCF). Cells were washed once with SOT medium at room temperature then resuspended in 2 ml of SOT. A 30-μl aliquot of cells was mixed with 300 ng of plasmid DNA and incubated at room temperature for 3 h. Samples were transferred to 0.6 ml of SOT medium in 14-ml round-bottom tubes and incubated overnight at 25–30 °C under 50–100 μEi of fluorescent light on a light rack. Each tube received 2.4 ml of SOT with appropriate antibiotics and was incubated under Multitron conditions to start selection. For the first 20–30 days, culture medium was changed every 3–5 days. After 30 days, when the transformants were robustly growing, cells were diluted every 3–5 days to facilitate segregation.

### Genotyping of transformed spirulina

Genomic DNA was prepared from spirulina cells by digestion with proteinase K. In brief, 0.2–0.5 OD_700_ of cells was washed once with sterilized water. A 30-μl sample of cell pellet was mixed with 120 μl of buffer EB (10 mM Tris-Cl pH 8.5). Proteinase K was added to samples at a final concentration of 0.2 mg ml^–1^. Samples were incubated at 56 °C for 1 h followed by 95 °C for 10 min, to deactivate proteinase K. Samples were centrifuged briefly in a microfuge to pellet cell debris. A 1-μl sample of the supernatant was used per genotyping PCR reaction. Specific integration of the transgenic cassette was determined by separate PCRs for each homology arm. For each PCR, one primer annealed to a genomic sequence outside the homology arm and the other to a region within the transgene. Segregation of the chromosome was assessed using a primer pair annealing to regions within the RHA and LHA. Segregation PCR yielded fragments of two different sizes: one from the wild-type allele and the other from the transgenic allele. Strains were considered fully segregated when no wild-type allele amplicon was observed.

To verify the sequence of the transgene, PCR was performed with genomic DNA to amplify the fragments, which includes the transgene, the homology arms and 500 bp flanking each homology arm. PCR products were separated by electrophoresis on an agarose gel, and amplified bands were gel extracted using the Qiagen Gel Extraction kit. Purified PCR products were sequenced to verify the integrated gene and surrounding sequences.

To exclude the possibility of cross-contamination with other strains, PCR was performed to check other loci used for integration of exogenous genes. PCR of genomic DNA using locus-specific primers was performed, and fragment size was analyzed by agarose gel electrophoresis. DNA fragments were gel extracted and characterized by Sanger sequencing. A strain was considered free of other spirulina strains if only wild-type loci were observed. Once strains were homozygous, the DNA sequence of the transgene was periodically reassessed by Sanger sequencing of PCR products. No DNA sequence variation of the transgene was observed, even after 3 years of continuous cultivation.

### Transformation of barcoded integrating plasmids

To evaluate the number of individual successful integration events per transformation, a library of DNA barcodes was transformed into spirulina and quantified by next-generation sequencing (NGS). In brief, a 19 N barcode was cloned adjacent to an antibiotic marker (*aadA*) in a plasmid containing homology arms for integration. The barcode library was estimated to contain >8 × 10^7^ transformants. The library was transformed into strain SP3 in triplicate, following the transformation method described above, and cultured with streptomycin. Spirulina samples were collected 22 and 28 days after transformation. Genomic DNA was extracted from spirulina and used in a PCR reaction to prepare ~320-bp amplicons of the barcoded regions for NGS analysis on a MiSeq (Illumina). Sequencing reads were filtered for quality and analyzed to minimize false positives. Counting only barcodes that were (1) present at both time points within a replicate, (2) unique to each replicate and (3) observed >30 times within a replicate yielded an estimated number of integration events of ~100–300.

### Isolation of single spirulina filaments

From an actively growing spirulina culture, 200–500 filaments were spread on a SOT agar plate. Cells were allowed to settle on the plate for 1–2 h and examined under a dissection microscope. Well-separated single filaments were picked with a 1-ml pipette tip and transferred in 3 ml of SOT with appropriate antibiotics in a round-bottom tube. Typically, 10–20 single filaments were cultured in Innova for 15 days for propagation.

### Determination of transgene copy number

To assess the copy number of an integrated transgene, three sets of primer/taqman probe pairs were designed to target three regions: an endogenous spirulina gene present at a single locus (*cpcB*), a promoter region present at both an endogenous and transgenic locus (that is, two chromosomal copies) and an exogenous region unique to the transgene. A synthetic g-block containing the three target loci plus flanking sequences was purchased from IDT as a calibrator. Real-time PCR was performed with the above primer/probes using genomic DNA from the transgenic spirulina strain and the g-block as templates. As controls, the parental spirulina strain and a second transgenic strain lacking template for the transgene-specific probes were tested. The relative copy number of the integrated transgene was calculated as the fold difference between transgene and endogenous genes using the △△C_t_ method. The experiment was repeated five times with three separate preparations of genomic DNA. The expected abundance ratio for the endogenous gene, promoter and exogenous gene was 1, 2 and 1 respectively.

### Axenic strain isolation

To establish axenic spirulina strains, cells were washed with SOT medium on 10-μm filters to exclude small, unicellular bacteria, and single filaments were isolated from cells captured on the filter. Cells were grown to a density of 0.5–1.0 OD_750_ ml^–1^ in an Innova incubator with appropriate antibiotics. Cells were pelleted from 5-ml cultures by centrifugation for 10 min at 1,600× RCF. To maintain sterility, the following steps were performed in a laminar-flow hood. The cell pellet was resuspended in 1 ml of SOT and transferred to a 10-μm filter prewetted with SOT medium, which was removed by gravity filtration. Cells were washed with successive 1-ml aliquots of SOT medium until at least 200 ml of total medium had passed through the filter. The remaining cells were resuspended with 0.5 ml of SOT and transferred to a sterile Eppendorf tube. Filaments were counted under a microscope as above, and 200–500 were spread on a SOT agar plate. Single filaments were isolated as above. After ~15 days, 10 μl of culture was spread on LB plates without antibiotics. Plates were incubated for 3–5 days in an incubator at 37 °C. Filament cultures free of contaminants on the LB agar plates were then seeded in 10 ml of SOT with 2.5 g l^–1^ dextrose at a density of 0.1 OD_750_ ml^–1^. Cultures were grown in Multitron for 3 days. A 100-μl sample of the culture was plated on LB agar plates without antibiotics and incubated at 37 °C for 5 days. Cultures with no contaminants observed on either set of LB plates were considered axenic.

### Culture of non-spirulina microbes

To culture non-spirulina microbes, a flask of spirulina culture was placed on bench for 3–5 h to allow cells to settle at the bottom of the flask. A 100-μl sample of supernatant was carefully pipetted and transferred to either LB or mixed LB/SOT agar plates. Plates were incubated at 25–30 °C on a light rack (60–70 μEi) for 5–7 days. Single colonies were streaked on fresh plates for between five and ten rounds. Cells from single colonies were spread on fresh plates to propagate for further experiments.

### Identification of non-spirulina bacteria from spirulina cultures

To culture non-spirulina microbes, a flask of spirulina culture was placed on the bench for 3–5 h to allow cells to settle at the bottom of the flask. A 100-μl sample of spirulina-conditioned medium was transferred to either LB agar or mixed LB/SOT agar plates, which were then incubated at 25–30 °C on a light rack (60–70 μEi) for 5–7 days. Genomic DNA was extracted from bacterial samples following the extraction method described above. Highly conserved and degenerate 16 S and 23 S ribosomal DNA PCR primers (Supplementary Table 1) were used to amplify genomic DNA, following published protocols^50,51^ from samples derived from both LB and LB/SOT plates. PCR product libraries were subcloned and sequenced. The probable species of origin was identified by BLAST query for similar sequences in the NCBI database.

### Markerless strain engineering

To create a platform for markerless integration, a parental strain containing a recombinant, non-native antibiotic marker was first generated. An integrating plasmid bearing homology arms for the D01030 (*kmR*) locus flanking an *aadA* gene for streptomycin resistance was transformed into wild-type spirulina. The integrating vector was designed to precisely replace the open reading frame (ORF) of D01030 with the sequence for *aadA*. This vector was transformed into spirulina strains UTEX (SP3) and NIES (SP7), generating strains SP205 and SP207, respectively. After transformation, strains were propagated for 2 months and confirmed to be fully segregated by genotyping. The strains were also challenged with kanamycin to demonstrate loss of native kanamycin resistance.

### Verification of markerless spirulina strains

Clonal isolates of fully segregated strains were verified as follows: (1) qPCR to demonstrate a single transgene per genome (above); (2) sequencing of chromosomal DNA to verify the absence of mutations in the homology arms and inserted gene(s) (above); (3) PCR to demonstrate loss of parental integration locus allele and complete segregation to homozygosity of the transgene (above); (4) chromosome DNA sequence of the 16 S rDNA locus to verify strain identity as *A. platensis* (above); (5) sequencing of alternative insertion sites in chromosomal DNA to verify lack of strain contamination with other engineered spirulina strains (above); (6) PCR to demonstrate absence of the integrating DNA vector backbone, which should be eliminated during integration by homologous recombination (below); and (7) verification of streptomycin sensitivity and kanamycin resistance by antibiotic challenge.

The vector backbone sequences outside of the homology arms should not integrate into the genome and thus should be absent from spirulina genomic DNA. To exclude the possibility of nonspecific integration of the vector backbone DNA, PCR was performed with primer pairs targeting the ampicillin resistance gene and *E. coli* origin of replication. At no point were these fragments observed in spirulina, suggesting that there is no integration of the vector outside of the homology arms.

### Construction of markerless transgenes for spirulina integration

To ease cloning of transgenes into spirulina, a standardized vector was built for markerless integration. This ‘destination’ vector included integrating homology arms for the *kmR* locus flanking an ORF for the native *kmR* gene and a terminator. The antibiotic marker was followed by a recombinant promoter–terminator pair for transgene expression. The promoter–terminator pair consisted of a constitutively active, native *A. platensis* promoter (600 bp upstream of the *cpcB* gene, named Pcpc600) and the terminator of the *E. coli* ribosomal RNA gene *rrnB* (named TrrnB). A pair of *Batrachochytrium salamandrivorans* restriction endonuclease sites between the promoter–terminator pair was used for Golden Gate cloning of protein coding sequences for transgenic expression. Protein coding sequences with compatible *B. salamandrivorans* sites were purchased from IDT and cloned into the destination vector using a Golden Gate Assembly Kit (NEB). Plasmid DNA was purified from *E. coli* by the QIAprep Spin Miniprep Kit (Qiagen) and transformed into spirulina strain SP205. The product of integration of this construct is genetically identical to the wild-type *kmR* locus, excepting the transgene (that is, no non-native antibiotic markers are present).

### Purification of recombinant protein from spirulina

Recombinant aa682 was purified from spirulina by immobilized metal affinity chromatography (IMAC). In brief, a 10-ml pellet of spirulina cells from strain SP1182 was collected from 2 l of culture by centrifugation. The pellet was resuspended in a total volume of 35 ml with lysis buffer (50 mM sodium phosphate buffer pH 8.0, 500 mM NaCl, 20 mM imidazole) supplemented with Pierce Protease Inhibitor Minitablets (Thermo Scientific) and 1 mM phenylmethylsulfonyl fluoride (PMSF). The resuspension was passed through a French pressure cell twice to lyse the cells. Samples were kept on ice throughout. The insoluble fraction was pelleted by centrifugation at 5,000× RCF for 30 min. The partially clarified lysate was mixed with 2 ml of washed HisPur Ni-NTA Resin (Thermo Scientific) and incubated at 4 °C with gentle rocking for 2 h. The resin was gently pelleted by centrifugation at 500× RCF for 1 min, supernatant discarded and the resin resuspended in fresh lysis buffer. This process was repeated until the supernatant was clear. The resin was collected in a small column by gravity filtration, washed with 20 ml of lysis buffer and spirulina-expressed aa682 was eluted with lysis buffer containing 200 mM imidazole. Purified aa682 was further polished by separation on a Superdex 75 Prep Grade column on an ÄKTA Pure, yielding a single band by SDS–polyacrylamide gel electrophoresis (SDS–PAGE) electrophoresis.

### Preparation of spirulina lysates for analysis of soluble protein

Soluble lysates from spray-dried spirulina samples were prepared using a flash-freeze protocol. Dried spirulina biomass was resuspended in PBS containing Pierce Protease Inhibitor minitablets and 1 mM PMSF at a biomass concentration of 10–40 mg ml^–1^ in 1.7-ml Eppendorf tubes. Samples were mixed to resuspend biomass powder and flash-frozen in liquid nitrogen for 2–5 min. Resuspensions were transferred to a water bath at 37 °C for 2–10 min. Samples were well mixed by inversion once thawed. The flash-freeze procedure was repeated an additional two times. Biomass samples were then centrifuged at 15,000*g* at 4 °C for 15–30 min, and the soluble fraction was transferred to a separate tube for downstream applications.

### Expression analysis of recombinant proteins in spirulina

Recombinant protein expression in spirulina was measured by capillary electrophoresis immunoassay (CEIA) using a Jess system (ProteinSimple), which was run as recommended by the manufacturer. In brief, dried biomass samples were diluted to a concentration of 0.2 mg ml^–1^ using water and a 5× master mix prepared from an EZ Standard Pack 1, in either reducing or nonreducing format (Bio-Techne). Purified protein controls used to generate standard curves were typically loaded at a range of concentrations from 0.5 to 20 μg ml^–1^. A 12–230-kDa Jess/Wes Separation Module (ProteinSimple) was used and 3 μl of each sample was loaded for 9 s. A mouse anti-His-tag antibody (GenScript) was diluted 1:100 and used as the primary detection antibody. An anti-mouse horseradish peroxidase (HRP)-conjugated secondary antibody (ProteinSimple) was primarily used for chemiluminescent detection; fluorescently labeled anti-mouse antibodies (ProteinSimple) for infrared or near infrared fluorescence detection were used for some experiments. Data analysis was performed using the Protein Simple Compass software.

### Expression, purification and biotinylation of *E. coli*-expressed proteins

Recombinant *C. jejuni* flagellin was expressed and purified from *E. coli*. A region of FlaA (sequence ID: WP_178888959.1) predicted to be soluble and exposed on the surface of flagella (amino acids 177–482) was cloned onto the C terminus of MBP in a pET28b *E. coli* expression vector. The vector was transformed into BL-21(DE3) cells and grown overnight at 37 °C on agar plates with kanamycin, and a single colony was used to inoculate a culture of LB medium containing kanamycin. Cells were grown overnight with shaking at 225 r.p.m. and 37 °C, back diluted to OD_600_ = 0.05 and grown at 37 °C until cells reached mid-log phase (OD_600_ = 0.4–0.6). Cells were induced with sopropyl-β-d-thiogalactoside and incubated with shaking at 16 °C overnight. The following day, cells were pelleted by centrifugation at 3,500× RCF for 20 min at 4 °C, resuspended in 30 ml of lysis buffer containing protease inhibitors and lysed in a Q700 Sonicator (Qsonica). The MBP–FlaA fusion was purified from the clarified lysate using Amylose Resin (NEB) according to the manufacturer’s recommendations, and purified protein was aliquoted and stored at –80 °C. Biotinylated MBP–FlaA protein was prepared using an EZ-Link NGS-PEG4-Biotin kit (Thermo Scientific) following the manufacturer’s guidelines.

VHHs expressed in *E. coli* used similar expression vectors and bacterial cells lines. Culturing, induction and lysis of *E. coli* expressing VHHs followed the same protocol as for FlaA expression. Purification of VHHs from lysates was performed by IMAC, following the purification protocol described for aa682.

The RBD antigen used with VHH-72 was kindly provided by R. Strong (Fred Hutchinson Cancer Institute).

### ELISA binding assays

The half-maximal effective concentration (EC_50_) binding activity of VHHs as a purified protein, and in spirulina lysate, was measured by ELISA. High-binding, 96-well plates (Greiner Bio-one or NUNC MaxiSorp) were coated with antigen by the addition of 100 μl of 1–5 μg ml^–1^ recombinant protein (FlaA or RBD antigen) in carbonate-bicarbonate buffer (Sigma) to each well and incubation overnight at 4 °C. Plates were washed three times with 300 μl of PBS supplemented with 0.05% Tween-20 (PBS-T). Washed plates were blocked with 250 μl of PBS-T supplemented with 5% nonfat dry milk (PBS-TM) for 2 h at room temperature. Blocking solution was discarded, and 100 μl of sample containing VHH was added to each well. VHH samples were prepared by dilution of purified protein or spirulina extracts with PBS-TM, and samples in a dilution series were serially diluted with PBS-TM. Samples were incubated at room temperature for 1 h to allow binding of VHH to antigen. After incubation, plates were washed three times with 300 μl of PBS-T 3. Wash was discarded, 100 μl of primary antibody diluted with PBS-TM was added to each well and plates were incubated at room temperature for 1 h. A 1:10,000 dilution of either a mouse anti-His-tag antibody (GenScript) or rabbit anti-camelid VHH antibody cocktail (GenScript) was used as the primary antibody. After incubation, plates were washed three times with 300 μl of PBS-T, and 100 μl of a secondary antibody was added to each well. A 1:10,000 dilution of either HRP-conjugated goat anti-mouse antibody or HRP-conjugated donkey anti-rabbit antibody was used as the secondary antibody. Plates were incubated at room temperature for 30–45 min, then washed twice with PBS-T and once with PBS. Plates were developed using either a SeraCare KPL TMB Microwell Peroxidase Substrate System (Sera Care Life Sciences) or 1-Step Ultra TMB-ELISA Substrate Solution (Thermo Scientific) following the manufacturer’s recommendations. Peroxidase activity was quenched after 5–10 min with 50 μl of either 1 M HCl or 2 M sulfuric acid. Absorbance at 450 nm (A450) was measured on an M2 plate reader (Molecular Devices, SoftMax Pro software). All samples were tested in duplicate. Data analysis was performed using Prism (GraphPad Software).

### Kinetics binding analysis of VHHs

Kinetics binding measurements were performed by biolayer interferometry (BLI) using an Octet Red96e (Forte Bio). Biotinylated MBP–FlaA was loaded onto streptavidin biosensors at a loading concentration of 100 nM and loading time of 4 min. After loading, probes were allowed to reach a baseline equilibrium in kinetics buffer (PBS with 1% bovine serum albumin and 0.05% Tween-20) for 2 min. Association and dissociation were monitored for 20 and 140 s, respectively. Purified aa682 diluted with kinetics buffer was assayed at concentrations ranging from 1 μM to 10 nM; the 10-nM sample was excluded from analysis due to a weak signal. Two biosensors were used as references: a 0-nM aa682 control and a no-ligand control. Kinetics binding values were determined using Octet Data Analysis HT software (ForteBio). Curve fits were performed using a global fit across all concentrations of aa682 assuming a 1:1 binding model.

### Epitope mapping of VHH–antigen interaction

Epitope mapping of the interaction between FlagV6 and flagellin was performed using phage-displayed peptide fragments derived from a ~300-amino-acid-soluble fragment of *C. jejuni* FlaA. A sliding window of 30 amino acid fragments, with a two-amino-acid interval along the length of FlaA, was designed as oligos for cloning into the phagemid pADL-23c (Antibody Design Labs). The peptide library was cloned into the BglI site of the phagemid by Gibson Assembly and transformed into DH5a *E. coli*, yielding >6 × 10^4^ transformants. The phagemid library was cleaned up with QiaPrep Spin Minikit columns and transformed into electrocompetent TG1 cells (Lucigen). Phage production was induced with the pIII-deficient helper phage CM13d3 (Antibody Design Labs) to ensure polyvalent display of the peptide epitopes. Phage from an overnight culture in 2× YT medium was precipitated and washed following the manufacturer’s protocol. Wells of an ELISA plate were coated overnight with 100 μl of 1 μg ml^–1^ FlagV6 VHH in carbonate-bicarbonate buffer, washed with PBS-T and blocked with PBS-TM. The phage library was diluted with PBS-TM to a concentration of 10^12^ phages ml^–1^ and incubated at room temperature for 30 min. Phages were then panned for VHH binders by the addition of 100 μl of blocked phage to wells of the ELISA plate and incubation on a vibrating platform for 2 h at room temperature. Unbound phages were washed from wells with 6,300 μl of PBS-T. Bound phages were eluted at low pH by the addtion of 100 μl of 100 mM glycine pH 2.0 and incubation for 5 min with shaking. The elution buffer was neutralized with 40 μl of 2 M Tris pH 7.5 and used to reinfect phage-competent TG1 cells (Antibody Design Labs). Library amplification and panning were performed for two additional rounds. After the third round of panning, all phagemid-containing colonies were observed to contain the same peptide fragment by Sanger sequencing. Two independent replicates of the experiment yielded overlapping fragments that mapped to the D3 domain of flaA.

### Flow cytometry of VHH binding to *C. jejuni*

Binding of spirulina-expressed VHHs to a pure culture of *C. jejuni* was measured by flow cytometry. An aliquot of lysate prepared from spray-dried spirulina biomass was incubated with an equivalent volume of 10^7^ CFU ml^–1^ *C. jejuni* 81–176 for 1 h at 4 °C. After washing with PBS containing 2% fetal bovine serum (FBS), bacteria were incubated for 30 min with the anti-His-tag antibody (iFluor647, GenScript). Samples were washed with PBS containing 2% FBS, resuspended in 2% paraformaldehyde and acquired on an LSR Fortessa flow cytometer (BD Biosciences) using forward and side scatter parameters in logarithmic mode. Data were analyzed using either FlowJo (TreeStar) or FACS Diva software (BD Biosciences).

### Motility inhibition assay

The motility-inhibitory activity of spirulina-expressed aa682 was measured by the motility of *C. jejuni* through soft agar. All *C. jejuni* cultures were performed in a tri-gas incubator at 40 °C under microaerobic conditions (5% O_2_, 10% CO_2_) unless otherwise stated. Glycerol stocks of *C. jejuni* were first streaked on Campy Blood Agar Blaser plates (Thermo Scientific) and grown for 48 h. Bacteria were then used to inoculate 0.4% soft agar Mueller–Hinton (MH) plates by stab and incubated for 48 h. A slice of agar from the leading edge of motility halos was used to inoculate 10 ml of MH broth. Liquid cultures were incubated under standard conditions for 72 h. A 20-μl spot of 5 mg ml^–1^ purified aa682 in PBS was added to the center of soft agar MH plates and allowed to fully adsorb into the agar. VHH spots were inoculated with 1 μl of OD_600_ = 0.03 of *C. jejuni* from the liquid culture. Samples and controls were set up in triplicate. Plates were incubated under standard conditions. The diameter of motility halos was periodically measured and used to calculate area.

### Midscale production of spirulina biomass for preclinical trials

To prepare biomass for preclinical mouse trials, the scale of spirulina culture was increased and harvested biomass was spray-dried. Spirulina cultures were initially propagated in shake flasks in medium based on the standard cyanobacterial SOT medium under Multitron conditions. Shake flask cultures were used to inoculate airlift reactors, with medium modified by partial replacement of sodium bicarbonate with sodium carbonate such that initial culture pH was 9.8. Cells were grown at light levels of 500–2,500 μmol m^–2^ s^–1^, with temperature maintained at 35 °C. As the culture utilizes CO_2_ and grows, pH rises and thus CO_2_ is added to the airlift stream to maintain pH between 9.8 and 10. Cultures were inoculated at a concentration of 0.1–0.5 g l^–1^ biomass by ash-free dry weight, and harvested by filtration at 2–4 g l^–1^.

To prepare for spray-drying, the harvested biomass was rinsed with a dilute (0.1%) trehalose solution to remove excess media salts, concentrated again by filtration and then spray-dried in a centrifugal nozzle spray-dryer. Feed rate, air flow and inlet air temperature were controlled to maintain an outlet air temperature of 68–72 °C at the powder-separation hydrocyclone. Once collected from the hydrocyclone, the powder was sealed and stored in airtight, opaque mylar bags to prevent exposure to moisture or light. The powder was stored at room temperature.

Before use in animal trials, spirulina biomass was analyzed to confirm strain identity. Dried biomass was genotyped to confirm the presence of the correct transgene and the absence of contaminating sequences (above). CEIA and ELISA binding assays (above) were also performed to confirm expression and binding activity of spirulina-expressed VHH.

### Prophylactic treatment of *C. jejuni* infection in two mouse models

Two independent mouse models were used to test the efficacy of spirulina-expressed VHHs in the treatment of *C. jejuni* infection. Animal experiments at the University of Virginia were performed according to institutional review board (IRB) protocols. Animal experiments performed at the IRB were in accordance with the Swiss Federal Veterinary Office guidelines and authorized by the Cantonal Veterinary Office. In a pilot experiment with the first model of *C. jejuni* infection, 2–4-week-old C57BL/6 male mice were fed a zinc-deficient diet^52^ before challenge. Animals were maintained according to institutional protocols and fed a regular diet with ad libitum water for 3 days. Animals were then started on the study diet for 10 days, after which water was replaced by water containing an antibiotic cocktail for 3 days to condition gut flora for *C. jejuni* colonization. Water was replaced with untreated, antibiotic-free water for 1 day before *C. jejuni* challenge. On day 0, mice were given an inoculum of 10^6^ live *C. jejuni* cells (strain 81–176, resuspended in PBS) by oral gavage. Food and water were provided ad libitum throughout. Mice were given five doses of a spirulina resuspension before and after challenge. Spray-dried spirulina biomass was resuspended in PBS at a concentration of 10% (w/v). A 200-μl resuspension was delivered by oral gavage on days –1, 0, 1, 2 and 3 relative to challenge. Groups received either PBS (eight mice), SP227 treatment (four mice) or SP526 treatment (eight mice). Day-of-challenge dosing was administered 60 min before challenge. Food and water were withdrawn 30 min before treatment, then provided ad libitum. To assess efficacy, mice were monitored for symptoms of diarrhea, changes in weight and bacterial shedding in stool. Weight measurements were made daily for 7 days. Stool samples were collected on days 1, 3 and 7 post challenge.

A second experiment using the first model of infection involved a change of study diet and a reduced spirulina dose. Animals were fed a regional basic diet for 10 days, followed by 3 days of antibiotic treatment. Untreated water was provided for 1 day before *C. jejuni* challenge. On day 0, mice were given an inoculum of 10^6^ live *C. jejuni* cells (strain 81–176, resuspended in PBS) by oral gavage. A control group of four mice received no *C. jejuni* (PBS only). Food and water were provided ad libitum throughout. Mice were given three doses of spirulina before and after challenge. On days –1, 0 and 1 relative to challenge, mice were orally gavaged with 200 μl of spirulina resuspension or control. Day-of-challenge dosing was administered 60 min before challenge. Food and water were withdrawn 30 min before treatment, then provided ad libitum. Spirulina resuspension was prepared at a concentration of 2% (w/v) in PBS. Groups of mice were treated with either PBS (eight mice), SP257 biomass (four mice) or SP526 biomass (four mice). To assess efficacy, mice were monitored for changes in weight, bacterial shedding in stool and levels of biomarkers in cecum. Weight measurements were made daily for 7 days. Stool samples were collected on days 2, 4, 6, 8 and 10 post challenge. On day 11, levels of LCN-2 and MPO were measured in stool and cecal contents by ELISA (DuoSet ELISA Mouse Lipocalin-2/NGAL, R&D Systems).

In the second model of *C. jejuni* infection, mice were orally treated with a range of spirulina concentrations to identify the minimally effective prophylactic dose of therapeutic. Three-week-old C57BL/6 female mice were housed, five per cage, under standardized conditions (20 ± 2 °C, 55 ± 8% relative humidity, 12/12-h light/dark cycle). Food and water were available ad libitum and mice were monitored daily. Mice were pretreated orally with 10 mg of vancomycin in 200 μl of PBS at 48, 24 and 12 h before spirulina administration. A single 400-μl dose of spray-dried spirulina resuspended in PBS was administered by oral gavage to mice 1.5 h before infection with *C. jejuni* 81–176 (10^8^ CFU 200 μl^–1^ PBS). To monitor efficacy, mice were observed daily and stools were collected at 24, 48 and 72 h post infection. To monitor pathogen load, stools were resuspended and plated on MH agar plates containing 10 μg ml^–1^ vancomycin and trimethoprim.

Cecal polymorphonuclear neutrophils (PMNs) were measured by flow cytometry 72 h post infection. Mice were sacrificed and the cecum was removed, opened longitudinally, carefully separated from cecal content and washed twice with ice-cold PBS. The cecum was digested twice with RPMI and EDTA 5 mM for 30 min at 37 °C. Filtrated fragments were then digested in RPMI 5% FBS, 1 mg ml^–1^ collagenase type II and 40 μg ml^–1^ DNase I for 40 min. The filtered suspension, containing cecum lamina propria cells, was centrifuged for 5 min at 300*g* and resuspended in RPMI complete medium. Single-cell suspensions from cecal lamina propria were stained with labeled antibodies diluted in PBS with 2% FBS for 20 min on ice. The following mouse antibodies were used: APC-conjugated anti-CD11b diluted 1:200 (Biolegend) and PE-conjugated anti-GR1 diluted 1:200 (TONBO Bioscience). Samples were acquired on an LSR Fortessa (BD Biosciences) flow cytometer. Data were analyzed using FlowJo or FACS Diva software.

The inflammation status of mice was evaluated by measurement of fecal LCN-2 levels in fecal supernatants by ELISA (DuoSet ELISA Mouse Lipocalin-2/NGAL, R&D Systems). In brief, feces collected at sacrifice were resuspended at 0.01 g 100 μl^–1^ PBS, centrifuged for 10 min at 17,000*g* and diluted before performing ELISA according to the manufacturer’s instructions.

### Large-scale, continuous culture of spirulina

Spirulina cultures were grown at large scale (250 l) in airlift reactors following protocols similar to the midscale reactors described above. Cultures were inoculated into the same media described above for midscale cultures, at a concentration of 0.1–0.5 g l^–1^ biomass by ash-free dry weight, grown under identical temperature and pH controls and harvested by filtration over stainless steel screens at 2–4 g l^–1^. A portion of the harvested culture was used to inoculate serial cultures, and the remaining harvested biomass was used for spray-drying as above. The dried powder was sealed and stored at room temperature in airtight, opaque mylar bags to prevent exposure to moisture or light.

Post collection, quality control of powder lots included determination of concentration of the 6x-His-tagged protein using CEIA performed on a Jess system (ProteinSimple). Specific ligand binding activity was determined on an Octet Red96e biolayer interferometry instrument (Forte Bio) using recombinant, biotinylated *C. jejuni* FlaA protein attached to streptavidin-coated biosensors. In addition, microbial characterization was performed with USP <61> and <62> and elemental impurities determined by USP <233>.

### Long-term stability of dried spirulina biomass

Batches of SP1182 spray-dried biomass were stored at room temperature and collectively assessed for binding activity by ELISA. Duplicate biomass samples from each batch were resuspended in PBS, lysed by freeze–thaw extraction and clarified by centrifugation. The binding activity of aa682 present in lysates was determined by ELISA with a recombinant FlaA antigen as described above. Purified aa682 was used to generate a standard curve for binding activity by linear regression using Excel (Microsoft software). The standard curve was used to calculate the concentration of aa682 in SP1182 lysates. The percentage of expected VHH activity was determined by normalization of aa682 concentration in each lysate to an assumed concentration of 3% aa682 per unit biomass.

### In vitro gastric protease digests of dried spirulina biomass

Spray-dried SP1182 biomass was exposed to simulated gastric fluid (SGF) to determine the stability of the aa682 present in spray-dried spirulina. A sample of spray-dried SP1182 biomass was resuspended in PBS at 30 mg ml^–1^. This resuspension was diluted 1:30 with prechilled SGF (50 mM citrate-phosphate buffer pH 3.0, 94 mM NaCl, 13 mM KCl pH 3.5 with 2,000 U ml^–1^ pepsin (MP Biomedicals)) and incubated in a water bath at 37 °C. Protease activity was neutralized by the addition of 50 mM NaOH and 1 mM PMSF. Samples were pelleted by centrifugation at 17,000*g* for 5 min. Biomass pellets were solubilized using 1× NuPAGE LDS sample buffer to a final biomass concentration of 1 mg ml^–1^ and heated at 90 °C on a heat block for 10 min. A similar process was used to assess the stability of purified aa682, omitting the centrifugation step.

The stability and activity of biomass-encapsulated aa682 after exposure to low-pH, simulated gastric buffer was assessed by CEIA and ELISA binding assay. Spray-dried SP1182 biomass was resuspended in either 50 mM bicarbonate buffer or citrate-phosphate buffer pH 3.0 with 1 mM PMSF. Samples were incubated in a water bath at 37 °C for 60 min. After incubation, biomass resuspensions were pelleted at 10,000 r.p.m. for 5 min. The supernatant was transferred to fresh tubes and stored at 4 °C. Pellets were resuspended in 1 ml of 50 mM bicarbonate buffer to a final biomass concentration of 30 mg ml^–1^ and incubated in a water bath at 37 °C for 30 min. Resuspensions were treated to three cycles of flash-freezing in liquid nitrogen, followed by thawing at 37 °C for extraction of soluble protein. After the final thawing, samples were pelleted using a refrigerated tabletop centrifuge for 30 min at 17,000*g* to separate soluble protein from insoluble cellular debris. The supernatant was used to measure the expression level and binding activity of aa682 by CEIA and ELISA, respectively.

### In vitro protease digests with intestinal proteases

To measure intestinal protease resistance, SP1182 lysates were digested with trypsin and chymotrypsin, and VHH binding activity was assessed by ELISA. Total soluble extract was prepared from a resuspension containing 40 mg of dried SP1182 biomass per 1 ml of bis-tris buffer (20 mM bis-tris pH 6.0) by the freeze–thaw protocol described above. Two volumes of soluble extract were mixed with one volume of protease in bis-tris buffer and one volume of PBS, to yield a final digest concentration of 0.1 or 0.01 mg ml^–1^ of trypsin or chymotrypsin with a reaction pH of ~6.5. Digests were performed at 37 °C for 1 h with shaking at 900 r.p.m. on an Eppendorf Thermomixer. Protease activity was neutralized by the addition of an equivalent volume of 2 mM PMSF and 2× Pierce Protease Inhibitor Mini tablets (Thermo Scientific) in PBS. Binding activity of VHH to recombinant FlaA was measured by ELISA as described above.

### Intact mass spectrometry

The mass of purified aa682 was analyzed with a 6230 TOF (Agilent) using an ACQUITY UPLC Protein BEH C4 VanGuard pre-column (Waters Corp.). The following settings were used: temperature, 30 °C; injection volume, 20 μl; mobile phase A of water with 0.1% formic acid (FA); mobile phase B of acetonitrile with 0.1% FA; sheath gas flow rate, 10 l min^–1^; sheath gas temperature, 350 °C; nebulizer pressure, 20 psig; gas flow rate, 10 l min^–1^; gas temperature, 325 °C; nozzle voltage, 2,000 V; V_cap_, 4,000 V; and mass range, 400–3,200 *m/z*.

### Peptide mapping by mass spectrometry

The peptide sequence of aa682 was confirmed by mass spectrometry of peptide fragments produced by protease digestion. A 50-μl sample of 1 mg ml^–1^ aa682 was reduced and denatured with 6 μl of 0.25 M DTT and 10 μl of 6 M guanidinium HCL for 30 min at 37 °C in the dark. The denatured sample was diluted with 60 μl of PBS and then digested with 12 μl of 1 mg ml^–1^ trypsin and chymotrypsin overnight at 37 °C. The digested product was prepared for LC–MS with 2 μl of 5% trifluoroacetic acid (TFA) and run on an UltiMate 3000 UHPLC system (Thermo Scientific) with an LTQ XL mass spectrometer (Thermo Scientific) using a CSH C18 Column (Waters Corp.) The following settings were used: temperature, 50 °C; injection, 40–50 μg sample; mobile phase A of water with 0.05% TFA; and mobile phase B of LC–MS acetonitrile with 0.05% TFA. Peptide mapping data were processed with *PepFinder 2.0* software (Thermo Scientific).

### First-in-human clinical trial

A phase 1 clinical trial was designed and conducted to assess the safety and tolerability of LMN-101. The study protocol and all its amendments were reviewed and approved by the Alfred Hospital Ethics Committee. Eligible, healthy volunteers aged 18–50 years were enrolled following informed consent. The study was performed in accordance with ICH guidelines and in compliance with all local and international requirements. Details of the study can be found at clinicaltrials.gov (ID: NCT04098263).

### Reporting Summary

Further information on research design is available in the Nature Research Reporting Summary linked to this article.

## Online content

Any methods, additional references, Nature Research reporting summaries, source data, extended data, supplementary information, acknowledgements, peer review information; details of author contributions and competing interests; and statements of data and code availability are available at 10.1038/s41587-022-01249-7.

## Supplementary Information

Supplementary Information Supplementary Table 3 and references.

Reporting Summary

Supplementary Tables Supplementary Table 1: Identification of non-spirulina bacteria. For each identified bacterial species the highest-identity BLAST hit is provided, as well as the relevant rDNA amplification primers and rDNA sequence. Supplementary Table 2: Characterized spirulina strains. Information on 30 spirulina strains referenced in this study is provided, including numbers of ORFs, introduced protein constructs, integration sites and antibiotic markers. Supplementary Table 4: Oligos used in this study. Nucleotide sequence, relevant figures and description of use are indicated.

## Figures and Tables

**Extended Data Fig. 7:**
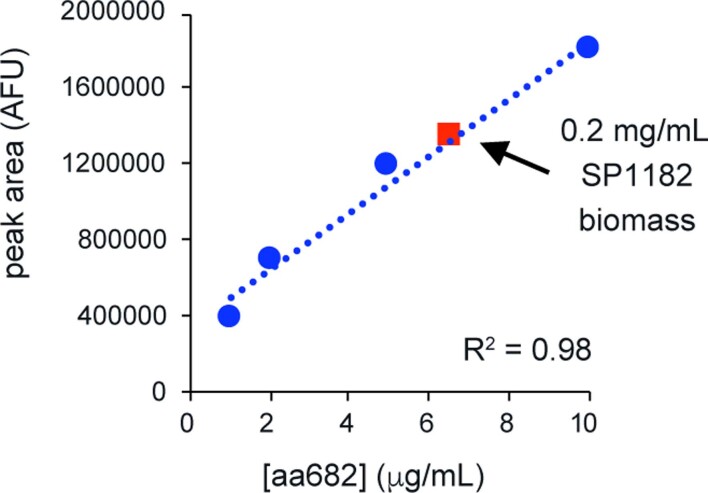
Linear regression analysis of CEIA standards for aa682 quantification in SP1182. A reference standard curve of purified aa682 protein measured on a Jess system by anti-His-tag detection is shown. Clarified lysate from spray dried SP1182 was loaded at a concentration of 0.2 mg biomass/mL. Using the standard curve, soluble recombinant aa682 was measured at ~3% of total dried biomass.

## Data Availability

Source data for the findings of this study will be made available on FigShare. Additional information on the strains reported here can be found in Supplementary Table 2. Due to some results containing unpublished proprietary information, these will be available from the corresponding author upon reasonable request.
