# Author Correction: Development of spirulina for the manufacture and oral delivery of protein therapeutics

**DOI:** 10.1038/s41587-022-01323-0

**Published:** 2022-04-19

**Authors:** Benjamin W. Jester, Hui Zhao, Mesfin Gewe, Thomas Adame, Lisa Perruzza, David T. Bolick, Jan Agosti, Nhi Khuong, Rolf Kuestner, Caitlin Gamble, Kendra Cruickshank, Jeremy Ferrara, Rachelle Lim, Troy Paddock, Colin Brady, Stacey Ertel, Miaohua Zhang, Alex Pollock, Jamie Lee, Jian Xiong, Michael Tasch, Tracy Saveria, David Doughty, Jacob Marshall, Damian Carrieri, Lauren Goetsch, Jason Dang, Nathaniel Sanjaya, David Fletcher, Anissa Martinez, Bryce Kadis, Kristjan Sigmar, Esha Afreen, Tammy Nguyen, Amanda Randolph, Alexandria Taber, Ashley Krzeszowski, Brittney Robinett, David B. Volkin, Fabio Grassi, Richard Guerrant, Ryo Takeuchi, Brian Finrow, Craig Behnke, James Roberts

**Affiliations:** 1 Lumen Bioscience, Seattle, WA USA; 2 Institute for Research in Biomedicine, Faculty of Biomedical Sciences, Università della Svizzera Italiana, Bellinzona, Switzerland; 3 Division of Infectious Diseases & International Health, University of Virginia School of Medicine, Charlottesville, VA USA; 4 Department of Pharmaceutical Chemistry, Vaccine Analytics and Formulation Center, University of Kansas, Lawrence, KS USA; 5 Present Address: Merck & Co., West Point, PA USA; 6 Present Address: Sandia National Laboratories, Livermore, CA USA; 7 Present Address: TScan Therapeutics, Waltham, MA USA

**Keywords:** Genetic engineering, Recombinant protein therapy, Expression systems, Protein delivery

Correction to: *Nature Biotechnology* 10.1038/s41587-022-01249-7, published online 21 March 2022.

In the version of this article initially published, the surname of author Bryce Kadis was misspelled as Kaldis. The name has been corrected in the HTML and PDF versions of the article.

