# Supplementary material for: Development of spirulina for the manufacture and oral delivery of protein therapeutics
Source: Nat Biotechnol. 2022 Mar 21;40(6):956–64. doi: 10.1038/s41587-022-01249-7 (PMC9200632; doi:10.1038/s41587-022-01249-7)
Supplement: Supplementary file 1 — Supplementary Table 3 and references. [file 41587_2022_1249_MOESM1_ESM.pdf]

nature portfolio

https://doi.org/10.1038/s41587-022-01249-7

Supplementary information

Development of spirulina for the manufacture and oral delivery of protein therapeutics

In the format provided by the authors and unedited

Preferred Term (PT)	LMN-101 Overall (N=15) n (%)	Placebo (N=6) n (%)	Overall (N=21) n (%)
**Subjects with at least one TEAE**	**5 (33.3%)**	**3 (50.0%)**	**8 (38.1%)**
Abdominal pain	1 (6.7%)	0	1 (4.8%)
Abdominal pain lower	1 (6.7%)	0	1 (4.8%)
Constipation	1 (6.7%)	0	1 (4.8%)
Diarrhea	1 (6.7%)	0	1 (4.8%)
Gastrointestinal sounds abnormal	0	1 (16.7%)	1 (4.8%)
Gastroesophageal reflux disease	1 (6.7%)	0	1 (4.8%)
Nausea	1 (6.7%)	0	1 (4.8%)
Vessel puncture site bruise	1 (6.7%)	0	1 (4.8%)
Lower respiratory tract infection viral	1 (6.7%)	0	1 (4.8%)
Pharyngitis	1 (6.7%)	0	1 (4.8%)
Viral infection	0	1 (16.7%)	1 (4.8%)
Viral upper respiratory tract infection	1 (6.7%)	0	1 (4.8%)
Headache	0	1 (16.7%)	1 (4.8%)
Anxiety	0	1 (16.7%)	1 (4.8%)
Menstruation delayed	1 (6.7%)	0	1 (4.8%)

Supplemental Table S3. Frequency and type of treatment-associated events in LMN-101 and placebo treated patients during the 29-day trial. All events were considered mild (grade 1). Evaluation by the trial physician indicated that none of these mild events were probably associated with treatment. Shown are the number of patients reporting an event (n), and the percentage of patients reporting an event (%).

Supplemental references

53. Taton, A. *et al.* The circadian clock and darkness control natural competence in cyanobacteria. *Nature Communications* 11, 1099–11 (2020).

54. Wrapp, D. *et al.* Structural Basis for Potent Neutralization of Betacoronaviruses by Single-Domain Camelid Antibodies. *Cell* 181, 1436–1441 (2020).

55. Zimmermann, I., Egloff, P., Hutter, C. & Elife, F. A. Synthetic single domain antibodies for the conformational trapping of membrane proteins. *elifesciences.org* doi:10.7554/elife.34317.001.

56. Burgers, P. P. *et al.* Structure of smAKAP and its regulation by PKA-mediated phosphorylation. *FEBS J* 283, 2132–2148 (2016).

57. Correnti, C. E. *et al.* Engineering and functionalization of large circular tandem repeat protein nanoparticles. *Nat Struct Mol Biol* 27, 1–20 (2020).

58. Hofmeyer, T. *et al.* Arranged Sevenfold: Structural Insights into the C-Terminal Oligomerization Domain of Human C4b-Binding Protein. *Journal of Molecular Biology* 425, 1302–1317 (2013).

59. Garaicoechea, L. *et al.* Llama-Derived Single-Chain Antibody Fragments Directed to Rotavirus VP6 Protein Possess Broad Neutralizing Activity In Vitro and Confer Protection against Diarrhea in Mice. *J. Virol.* 82, 9753–9764 (2008).

60. Koromyslova, A. D. & Hansman, G. S. Nanobodies targeting norovirus capsid reveal functional epitopes and potential mechanisms of neutralization. *PLoS Pathog* 13, e1006636 (2017).

61. Yang, Z. *et al.* A novel multivalent, single-domain antibody targeting TcdA and TcdB prevents fulminant Clostridium difficile infection in mice. *J. Infect. Dis.* 210, 964–972 (2014).
