# Supplementary material for: Development of spirulina for the manufacture and oral delivery of protein therapeutics
Source: Nat Biotechnol. 2022 Mar 21;40(6):956–64. doi: 10.1038/s41587-022-01249-7 (PMC9200632; doi:10.1038/s41587-022-01249-7)
Supplement: Supplementary file 2 — Reporting Summary [file 41587_2022_1249_MOESM2_ESM.pdf]

nature research

Corresponding author(s): James Roberts

Last updated by author(s): Feb 1, 2022

Reporting Summary

Nature Research wishes to improve the reproducibility of the work that we publish. This form provides structure for consistency and transparency in reporting. For further information on Nature Research policies, see our Editorial Policies and the Editorial Policy Checklist.

Statistics

For all statistical analyses, confirm that the following items are present in the figure legend, table legend, main text, or Methods section.

n/a	Confirmed	
☐	☒	The exact sample size (*n*) for each experimental group/condition, given as a discrete number and unit of measurement
☐	☒	A statement on whether measurements were taken from distinct samples or whether the same sample was measured repeatedly
☐	☒	The statistical test(s) used AND whether they are one- or two-sided<br>*Only common tests should be described solely by name; describe more complex techniques in the Methods section.*
☒	☐	A description of all covariates tested
☒	☐	A description of any assumptions or corrections, such as tests of normality and adjustment for multiple comparisons
☐	☒	A full description of the statistical parameters including central tendency (e.g. means) or other basic estimates (e.g. regression coefficient) AND variation (e.g. standard deviation) or associated estimates of uncertainty (e.g. confidence intervals)
☐	☒	For null hypothesis testing, the test statistic (e.g. *F*, *t*, *r*) with confidence intervals, effect sizes, degrees of freedom and *P* value noted<br>*Give P values as exact values whenever suitable.*
☒	☐	For Bayesian analysis, information on the choice of priors and Markov chain Monte Carlo settings
☒	☐	For hierarchical and complex designs, identification of the appropriate level for tests and full reporting of outcomes
☒	☐	Estimates of effect sizes (e.g. Cohen's *d*, Pearson's *r*), indicating how they were calculated

Our web collection on statistics for biologists contains articles on many of the points above.

Software and code

Policy information about availability of computer code

Data collection	Compass for Simple Western (V5.0.10), SoftMax Pro (v2.1.28), ForteBio Data Acquisition (v11.1.2.24)
Data analysis	Microsoft Excel (v16.49), Prism (v9.1.1), ForteBio Data Analysis HT (v11.1.2.48), PepFinder (v2.0), FlowJo (v10.7.1), FACS Diva (v6.2), PyMol (v2.1)

For manuscripts utilizing custom algorithms or software that are central to the research but not yet described in published literature, software must be made available to editors and reviewers. We strongly encourage code deposition in a community repository (e.g. GitHub). See the Nature Research guidelines for submitting code & software for further information.

Data

Policy information about availability of data

All manuscripts must include a data availability statement. This statement should provide the following information, where applicable:

- Accession codes, unique identifiers, or web links for publicly available datasets
- A list of figures that have associated raw data
- A description of any restrictions on data availability

Source data for the findings of this study will be made available on FigShare. Additional information on the strains reported here can be found in Supplemental Table S2. Due to some results containing unpublished proprietary information, these will be available from the corresponding author upon reasonable request.

Field-specific reporting

Please select the one below that is the best fit for your research. If you are not sure, read the appropriate sections before making your selection.

☒ Life sciences ☐ Behavioural & social sciences ☐ Ecological, evolutionary & environmental sciences

For a reference copy of the document with all sections, see nature.com/documents/nr-reporting-summary-flat.pdf

Life sciences study design

All studies must disclose on these points even when the disclosure is negative.

Sample size	For in vitro protein characterization experiments were performed in duplicate or triplicate. This sample size was considered sufficient because the methods used tend to be highly reproducible. No statistical test was used to calculate in vitro sample size. In vivo animal experiments were performed with cohorts of either 4 or 5 mice. This group size was dependent on the number of animals permitted per cage at the institution performing the experiment. The total number of mice used for each experiment was limited by the resources of the researchers.
Data exclusions	No data were excluded.
Replication	For most in vitro protein and biomass characterization, individual proteins and spirulina strains were evaluated in one to two independent experiments. Because the methods used for this characterization (PCR, ELISA, SDS-PAGE, CEIA, etc.) are standard and well-developed, a single replicate was considered sufficient for high confidence in the results.<br>Animal and human trial experiments have not been replicated due to the high cost of these experiments.
Randomization	Animals purchased for pre-clinical studies were randomly assigned to treatment or control groups. Human study participants were randomly assigned to active or placebo groups.
Blinding	Investigators managing the animal trials were provided with spirulina strain numbers but were blinded to the identity of the strain used for each treatment or control group. Strain identity was unblinded after data collection and analysis. Human clinical trial staff were blinded to drug assignment (active or placebo).

Reporting for specific materials, systems and methods

We require information from authors about some types of materials, experimental systems and methods used in many studies. Here, indicate whether each material, system or method listed is relevant to your study. If you are not sure if a list item applies to your research, read the appropriate section before selecting a response.

Materials & experimental systems

n/a	Involved in the study	
☐	☒	Antibodies
☒	☐	Eukaryotic cell lines
☒	☐	Palaeontology and archaeology
☐	☒	Animals and other organisms
☐	☒	Human research participants
☐	☒	Clinical data
☒	☐	Dual use research of concern

Methods

n/a	Involved in the study	
☒	☐	ChIP-seq
☒	☐	Flow cytometry
☒	☐	MRI-based neuroimaging

Antibodies

Antibodies used	THE™ His Tag Antibody mAb, Mouse (Genscript Cat. No. A00186; clone id: 6G2A9)<br>THE™ His Tag Antibody [iFluor 647] mAb, Mouse (Genscript Cat. No. A01802; clone id: 6G2A9)<br>MonoRab™ Rabbit Anti-Camelid VHH Cocktail (Genscript Cat. No. A02014)<br>Goat anti-mouse IgG (H+L)-HRP (Bio-Rad Cat. No. 170-6516)<br>Donkey anti-rabbit IgG HRP (Santa Cruz Bio Cat. No. sc-2305)<br>Anti-mouse CD11B, APC-conjugated (Biolegend Cat. No. 101212; clone id: M1/70)<br>Anti-mouse Gr1, PE conjugated (TONBO Bioscience Cat. No. 50-5931-U100; clone id: RB6-8C5)
Validation	Primary and secondary antibodies were validated for use in ELISA and western blot by testing with appropriate negative and positive controls.<br>His Tag Antibody - manufacturer describes antibody as high affinity, with specificity for 6xHis, as well as 5xHis and 4xHis<br>Anti-Camelid VHH antibody - manufacturer describes antibody as a mixture of several monoclonal antibodies, and according to the manufacturer, "It has no cross-reactivity with mouse, rat, rabbit, goat or human immunoglobulins."<br>Goat anti-mouse - manufacturer notes antibody "is prepared from antisera raised in goats immunized with purified mouse IgG. Immunoaffinity chromatography procedures are used to isolate antibodies and to remove antibodies which cross react with human immunoglobulin."

	Donkey anti-rabbit - this antibody has been discontinued, but www.citeab.com denotes that this antibody has been reported in 56 citations (https://www.citeab.com/antibodies/3244172-sc-2305-donkey-anti-rabbit-igg-hrp)<br>Anti-mouse CD11B, APC-conjugated - manufacturer notes quality control testing by immunofluorescent staining with flow cytometric analysis and that antibody has been verified for immunocytochemistry<br>Anti-mouse Gr1, PE-conjugated - manufacturer notes application use for flow cytometry; antibody may be cross-reactive with Ly-6G (ie Gr1) and Ly-6C

Animals and other organisms

Policy information about studies involving animals; ARRIVE guidelines recommended for reporting animal research

Laboratory animals	In the first set of animal experiments, 2-4 week old C57BL/6 male mice were used. In the second set of animals experiments, 3 week old C57BL/6 female mice were used.
Wild animals	Study did not involve wild animals.
Field-collected samples	Study did not involve samples collected in the field.
Ethics oversight	Animal experiments at University of Virginia were performed per IRB protocols. Animal experiments performed at IRB were in accordance with the Swiss Federal Veterinary Office guidelines and authorized by the Cantonal Veterinary Office.

Note that full information on the approval of the study protocol must also be provided in the manuscript.

Human research participants

Policy information about studies involving human research participants

Population characteristics	Human research participants were male or female between the ages of 18 and 50 years, of general good health without significant medial illness.
Recruitment	Healthy volunteers were recruited from the community by media and from the existing clinical research center population of healthy volunteers, following informed consent.
Ethics oversight	The study protocol and all its amendments were reviewed and approved by the Alfred Hospital Ethics Committee.

Note that full information on the approval of the study protocol must also be provided in the manuscript.

Clinical data

Policy information about clinical studies

All manuscripts should comply with the ICMJE guidelines for publication of clinical research and a completed CONSORT checklist must be included with all submissions.

Clinical trial registration	NCT04098263
Study protocol	The full study protocol is available from the the corresponding author upon reasonable request.
Data collection	This study was conducted November 20, 2019 to April 21, 2020 at Q-Pharm Pty Ltd (Herston QLD, Australia).
Outcomes	Primary outcome measures were the rate of adverse events in LMN-101 subjects and tolerability of LMN-101 compared to placebo. Adverse events were graded according to severity and rates were compared between LMN-101 subjects and placebo subjects. Tolerability was assessed by the proportion of subjects completing study drug and remaining on study and free from possibly drug-related and dose-limiting serious adverse events. Secondary outcome measures were peak serum concentration, area under the curve in serum, and anti-drug antibodies in LMN-101 subjects. The secondary outcomes were assessed by sandwich ELISA.
